# Heart failure with reduced ejection fraction in Sweden: patient adherence and persistence to quadruple pharmacotherapy prescription

**DOI:** 10.1093/eurheartj/ehag199

**Published:** 2026-04-01

**Authors:** Felix Lindberg, Alicia Uijl, Lina Benson, Valeria Valente, Andrew J S Coats, Michael Böhm, Marco Metra, Stefano Masi, Lars H Lund, Giuseppe M C Rosano, Gianluigi Savarese

**Affiliations:** Department of Clinical Science and Education, Karolinska Institutet, Södersjukhuset, Stockholm, Sweden; Department of Clinical Science and Education, Karolinska Institutet, Södersjukhuset, Stockholm, Sweden; Department of Cardiology Amsterdam University Medical Center, Amsterdam Cardiovascular Sciences, University of Amsterdam, Amsterdam, The Netherlands; Department of Clinical Science and Education, Karolinska Institutet, Södersjukhuset, Stockholm, Sweden; Department of Medicine, Solna, Karolinska Institutet, Stockholm, Sweden; Department of Clinical Science and Education, Karolinska Institutet, Södersjukhuset, Stockholm, Sweden; Heart Research Institute, University of Sydney, Sydney, New South Wales, Australia; HOMICAREM (HOMburg Institute for CArdioREnalMetabolic Medicine) and Department of Medicine III, Saarland University, Homburg, Germany; Cardiology, ASST Spedali Civili, Department of Medical and Surgical Specialties, Radiological Sciences and Public Health, University of Brescia, Brescia, Italy; Department of Clinical and Experimental Medicine, University of Pisa, Pisa, Italy; Department of Medicine, Solna, Karolinska Institutet, Stockholm, Sweden; Department of Human Sciences and Promotion of Quality of Life, San Raffaele Open University of Rome, Rome, Italy; Cardiology, IRCCS San Raffaele, Rome, Italy; Department of Clinical Science and Education, Karolinska Institutet, Södersjukhuset, Stockholm, Sweden

**Keywords:** Implementation, Heart failure, HFrEF, Adherence, Persistence, Guideline-directed medical therapy

## Abstract

**Background and Aims:**

Real-world uptake of heart failure with reduced ejection fraction (HFrEF) treatments relies on physician prescriptions but also on patient adherence. This study assessed implementation of, patient adherence and persistence with quadruple HFrEF therapy in the contemporary setting, and associations with morbidity/mortality.

**Methods:**

Patients with HFrEF enrolled in the Swedish Heart Failure Registry between January 2016 and December 2023 were included. Using pharmacy refills, patients with ≥80% proportion of days covered were categorized as adherent and with dispensations at 12 ± 2 months post-index as persistent.

**Results:**

In 35 215 patients with HFrEF [median age 74 (interquartile range 64–80) years; 28% female], use of all four HFrEF foundational therapies increased between 2016 and 2023: in 2023, 93% used beta-blockers (BB), 95% renin–angiotensin–system inhibitors (RASi)/angiotensin receptor–neprilysin inhibitors (ARNi), 73% mineralocorticoid receptor antagonists (MRA), 83% sodium–glucose co-transporter 2 inhibitors (SGLT2i), and 60% quadruple therapy. The proportion of patients who were adherent and persistent, respectively, to BB was 95% and 90%, RASi/ARNi 95% and 89%, MRA 90% and 77%, SGLT2i 94% and 86%, and quadruple therapy 85% and 67%. Good adherence and persistence for BB, RASi/ARNi, and MRA and good adherence for SGLT2i were independently associated with lower heart failure hospitalization/cardiovascular death.

**Conclusions:**

By 2023, use of quadruple therapy reached ∼60%, being mostly limited by lower adoption of MRA (∼73%). Patient adherence to foundational therapies was high and appeared associated with better prognosis. These data suggest that improvements in the uptake of and adherence to quadruple HFrEF pharmacotherapy are achievable in real-word heart failure care, although suboptimal dosing and frequent discontinuations remain important areas of further implementation.

## Structured Graphical Abstract

**Structured Graphical Abstract:**
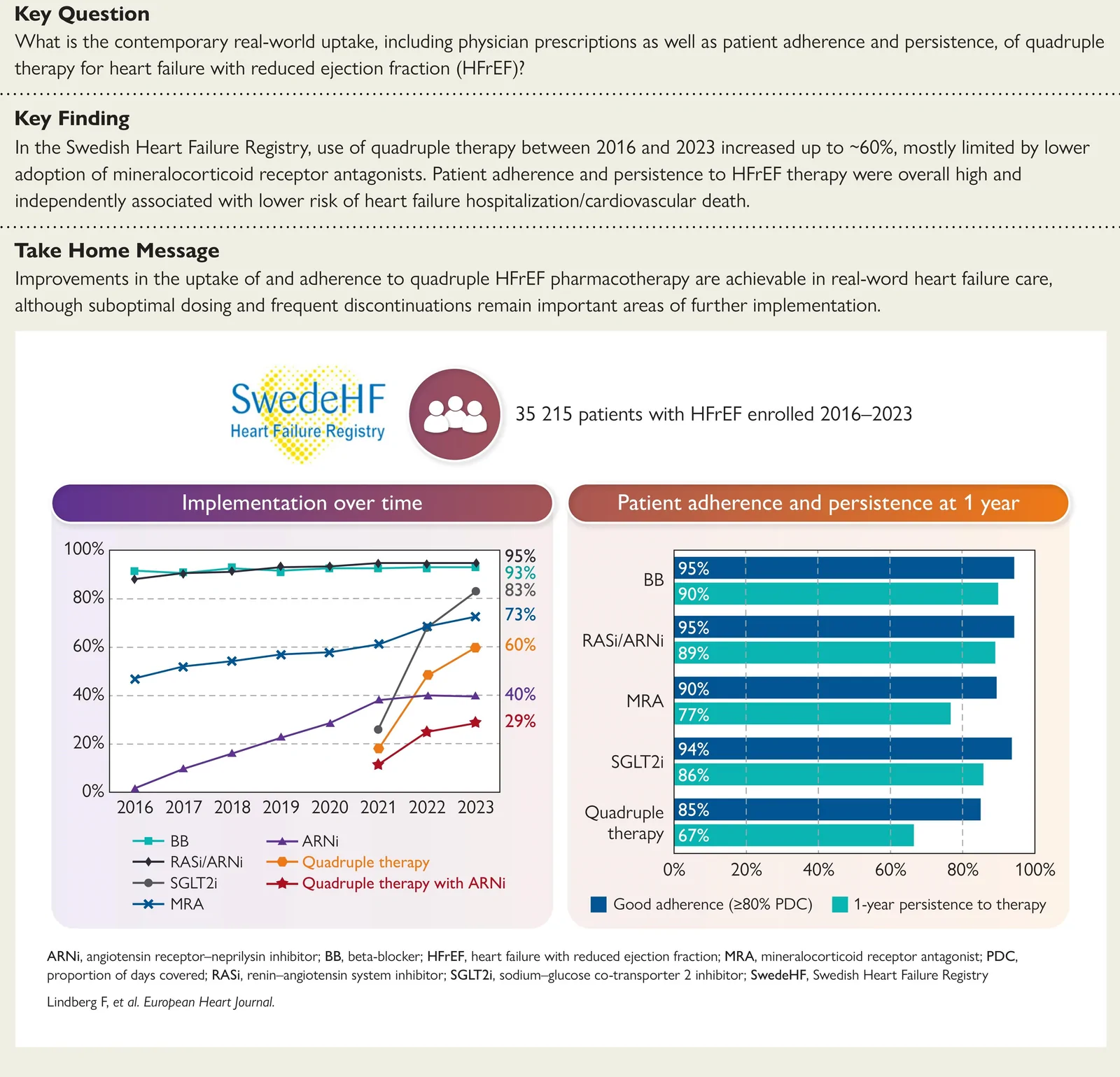

**See the editorial comment for this article ‘From prescription to persistence: guideline-recommended medical therapy in heart failure with reduced ejection fraction’, by J. Bauersachs and S. Soltani, https://doi.org/10.1093/eurheartj/ehag198.**

## Introduction

Quadruple therapy including a renin–angiotensin system inhibitor (RASi)/angiotensin receptor–neprilysin inhibitor (ARNi), beta-blocker, mineralocorticoid receptor antagonist (MRA), and sodium–glucose co-transporter 2 inhibitor (SGLT2i) is recommended for all patients with heart failure (HF) with reduced ejection fraction (HFrEF).^1^ The combined use of all four foundational therapies has been estimated to reduce hospitalizations for HF (HHF) or cardiovascular mortality by up to 64%.^2^ Real-world uptake of these therapies relies not only on physician prescriptions but also on patients actually taking the prescribed drugs (adherence) and continuing them over the longer term (persistence).^3^ Therefore, these factors inevitably affect how benefits of guideline-directed medical therapy (GDMT) achieved in trials are translated into real-world HFrEF populations.^4^ Although previous studies have highlighted limited implementation (physician prescriptions) of HF therapies,^5–8^ there is a lack of data on patient adherence and persistence to HF treatments, particularly in the era of quadruple foundational therapy.

The therapeutic toolkit for HFrEF has expanded in recent years, which might impact treatment uptake both in terms of implementation and patient adherence. Specific new therapies might facilitate uptake by improving clinical status and tolerability. For example, in the PARADIGM-HF and EMPEROR-Reduced trials, respectively, ARNi and SGLT2i were linked to less discontinuation of MRA.^9,10^ On the other hand, the increasing pill-burden and polypharmacy has been linked to lower patient adherence in the cardiovascular field.^11^

Therefore, in a large and contemporary HFrEF population, we performed a comprehensive assessment of the uptake of quadruple HFrEF pharmacotherapy, ranging from (i) physician prescriptions; (ii) patient adherence; and (iii) patient persistence, as well as associated patient characteristics and outcomes.

## Methods

### Data sources

Data from five Swedish registries were used for this study, interlinked via the unique personal identification number held by all Swedish residents. The study population was derived from the Swedish HF Registry (SwedeHF) which has been previously described.^12^ The inclusion criterion is clinician-judged HF, since 2017 defined by International Classification of Diseases, 10th Revision (ICD-10) codes reported in Supplementary data online, *Table S1*. In 2023, SwedeHF covered 59% of prevalent HF cases in Sweden.^13^ Pharmacy dispensations of prescribed drugs were extracted from the National Prescribed Drugs Register (NPDR), which is managed by the National Board of Health and Welfare, and collects information on all prescribed drugs [categorized according to Anatomical Therapeutic Chemical (ATC) codes] dispensed in Swedish pharmacies since July 2005. Socioeconomic variables were obtained through Statistics Sweden’s Longitudinal Integrated Database for Health Insurance and Labour Market Studies and the Register of the Total Population. The National Patient Register and Cause of Death Register, both managed by the National Board of Health and Welfare, provided additional comorbidity data, HHF, and cause-specific mortality. The cross-registry linkage and current analysis were approved by the Swedish Ethical Review Authority.

### Patients

Patients with HFrEF enrolled in SwedeHF between 1 January 2016 and 31 December 2023 were included. The index date was defined as the day of the outpatient visit or hospital discharge prompting the latest entry in SwedeHF. Heart failure with reduced ejection fraction was defined as ejection fraction (EF) < 40% rather than ≤40%, due to the categorization of SwedeHF-reported EF in most patients. Follow-up was censored at 3 years, 31 December 2023, death or move from Sweden. The patient selection flow chart is presented in Supplementary data online, *Table S2*.

### Variable definitions

Prescribed treatments at index were identified by pharmacy dispensations within 4 months prior until 14 days after the index date and assessed according to drug class and number of GDMT components (i.e. single, dual, triple, or quadruple therapy).

Prescribed daily dosages were calculated by multiplying the strength of the prescribed tablet with the number of pills per day prescribed, as algorithmically extracted from free text dose instructions reported in NPDR. For cases where prescribed dosages were missing or uninterpretable, the dose was imputed as the mean of non-missing dosages for the same ATC class. The daily dosage prescribed at the baseline dispensation was further quantified as the percentage of the substance-specific target dose recommended in the 2021 European Society of Cardiology HF guidelines (see Supplementary data online, *Table S3*).^1^

Proportion of days covered (PDC) was calculated for each dispensation interval (per medication class) by dividing the number of days covered (calculated as dispensed total dose divided by daily prescribed dosage) with the number of days between dispensations. Good adherence for each prescribed regimen was defined as ≥80% PDC during the first year post-index in the main analysis. The ≥80% PDC threshold was selected as it is well-established in pharmacoepidemiological studies.^14^ A sensitivity analysis was performed denoting good adherence instead as ≥88% PDC, based on a prior small study reporting this as the most prognostic cut-off in patients with HF.^15^

Patients filling prescriptions at 6 ± 2, 12 ± 2, 18 ± 2, and 24 ± 2 months post-index were considered persistent at 0.5, 1, 1.5, and 2 years, respectively. A 4-month time window (i.e. ± 2 months) was selected to add 1 month grace period (accounting for potential pill stockpiling) to the ∼3 months covered by the most commonly prescribed pill packages. Variable definitions, including ATC codes for prescribed treatments and ICD-10 codes for comorbidity variables and cause-specific outcomes, are reported in Supplementary data online, *Table S4*.

### Statistical analysis

Baseline characteristics were presented as median [interquartile range (IQR)] if continuous, and frequency (per cent) if categorical, and compared according to number of GDMT components at baseline by Kruskal–Wallis and *χ*^2^ test, respectively.

The proportion of patients being prescribed each therapy regimen was calculated overall and according to index year. For calculating the proportion of patients with good adherence and persistence, the denominator was the number of patients using the respective regimen at index (including prevalent as well as first-time users). For persistence at 0.5, 1, 1.5, and 2 years, patients also had to be alive at 6 + 2, 12 + 2, 18 + 2, and 24 + 2 months post-index. A sensitivity analysis was performed separately in first-time users, defined as patients using the respective regimen at index but without a prior prescription dispensation for that regimen in the past 12 months.

Patient characteristics associated with good adherence and 1-year persistence were assessed by multivariable logistic regression including variables reported in *Table 1*, with results reported as odds ratios (OR) with 95% confidence intervals (CI).

**Table 1:** Patient characteristics stratified by number of prescribed guideline-directed medical therapy components

Variable	Overall (*n* = 35 215)	None (*n* = 471, 1%)	Mono (*n* = 2093, 6%)	Double (*n* = 11 205, 32%)	Triple (*n* = 14 718, 42%)	Quadruple (*n* = 6728, 19%)	*P*-value	Missing (%)
Demographic characteristics								
Index year^a^	2020 [2018–2022]	2019 [2017–2021]	2018 [2017–2020]	2019 [2017–2021]	2019 [2018–2021]	2023 [2022–2023]	<.001	0
Index year							<.001	0
2016–20	19 966 (57)	348 (74)	1619 (77)	8403 (75)	9596 (65)	<10		
2021–23	15 249 (43)	123 (26)	474 (23)	2802 (25)	5122 (35)	6728 (100)		
Sex^a^							<.001	0
Female	9891 (28)	121 (26)	594 (28)	3346 (30)	4046 (27)	1784 (27)		
Male	25 324 (72)	350 (74)	1499 (72)	7859 (70)	10 672 (73)	4944 (73)		
Age (years)	74 [64–80]	73 [61–80]	78 [69–85]	75 [66–82]	73 [64–79]	72 [62–78]	<.001	0
Age (years)^a^							<.001	0
<70	13 053 (37)	208 (44)	524 (25)	3620 (32)	5737 (39)	2964 (44)		
70–80	13 785 (39)	148 (31)	733 (35)	4300 (38)	5917 (40)	2687 (40)		
>80	8377 (24)	115 (24)	836 (40)	3285 (29)	3064 (21)	1077 (16)		
Socioeconomic characteristics								
Family situation^a^							<.001	0
Cohabitating	19 033 (54)	194 (42)	1060 (51)	5944 (53)	8158 (56)	3677 (55)		
Living alone	16 139 (46)	271 (58)	1031 (49)	5252 (47)	6541 (44)	3044 (45)		
Marital status							<.001	0
Married	17 111 (49)	173 (37)	989 (47)	5441 (49)	7276 (49)	3232 (48)		
Single/widowed/divorced	18 061 (51)	292 (63)	1102 (53)	5755 (51)	7423 (51)	3489 (52)		
Children^a^	29 046 (82)	354 (75)	1755 (84)	9346 (83)	12 137 (82)	5454 (81)	<.001	0
Education^a^							<.001	1
Compulsory school	11 610 (33)	153 (34)	813 (40)	4048 (37)	4837 (33)	1759 (26)		
Secondary school	15 621 (45)	209 (46)	824 (40)	4807 (43)	6541 (45)	3240 (49)		
University	7500 (22)	93 (20)	411 (20)	2210 (20)	3138 (22)	1648 (25)		
Income^a^							<.001	0
1st tertile within year	11 598 (33)	205 (44)	738 (35)	3748 (33)	4753 (32)	2154 (32)		
2nd tertile within year	11 604 (33)	158 (34)	749 (36)	3858 (34)	4720 (32)	2119 (32)		
3rd tertile within year	11 970 (34)	102 (22)	604 (29)	3590 (32)	5226 (36)	2448 (36)		
Clinical characteristics								
Duration of HF (months)^a^							<.001	2
<6	16 553 (48)	153 (34)	786 (39)	5482 (50)	6496 (45)	3636 (55)		
≥6	17 882 (52)	296 (66)	1243 (61)	5410 (50)	7923 (55)	3010 (45)		
Previous HHF within 3 months^a^	11 325 (32)	170 (36)	632 (30)	3359 (30)	4991 (34)	2173 (32)	<.001	0
Previous HHF within 1 year	14 637 (42)	215 (46)	797 (38)	4196 (37)	6551 (45)	2878 (43)	<.001	0
Follow-up referral HF nurse clinic^a^	29 810 (88)	291 (71)	1515 (78)	9200 (86)	12 583 (89)	6221 (95)	<.001	4
Follow-up referral speciality^a^							<.001	2
Primary care/other	5208 (15)	140 (32)	656 (33)	2163 (20)	1787 (12)	462 (7)		
Hospital	29 203 (85)	297 (68)	1338 (67)	8758 (80)	12 642 (88)	6168 (93)		
LVEF, *n* (%)^a^							<.001	0
30%–39%	21 037 (60)	261 (55)	1427 (68)	7156 (64)	8433 (57)	3760 (56)		
<30%	14 178 (40)	210 (45)	666 (32)	4049 (36)	6285 (43)	2968 (44)		
NYHA class							<.001	18
I	3281 (11)	31 (10)	153 (10)	998 (11)	1315 (11)	784 (13)		
II	14 604 (50)	125 (42)	671 (46)	4436 (50)	6200 (50)	3172 (53)		
III	10 706 (37)	127 (42)	606 (41)	3364 (38)	4660 (38)	1949 (33)		
IV	410 (1)	18 (6)	40 (3)	134 (2)	172 (1)	46 (1)		
NYHA class^a^							<.001	18
I–II	17 885 (62)	156 (52)	824 (56)	5434 (61)	7515 (61)	3956 (66)		
III–IV	11 116 (38)	145 (48)	646 (44)	3498 (39)	4832 (39)	1995 (34)		
BMI (kg/m^2^)	26 [24–30]	26 [23–29]	26 [23–29]	26 [23–30]	27 [24–30]	27 [24–31]	<.001	22
BMI (kg/m^2^)^a^							<.001	22
<30	20 321 (74)	258 (78)	1213 (78)	6662 (76)	8456 (73)	3732 (71)		
≥30	7182 (26)	72 (22)	350 (22)	2147 (24)	3120 (27)	1493 (29)		
Systolic blood pressure (mmHg)	120 [110–135]	122 [107–140]	125 [112–140]	123 [110–139]	120 [110–135]	120 [105–130]	<.001	4
Systolic blood pressure (mmHg)^a^							<.001	4
100–139	23 582 (70)	280 (63)	1335 (67)	7379 (68)	9994 (70)	4594 (71)		
<100	3031 (9)	50 (11)	119 (6)	760 (7)	1340 (9)	762 (12)		
≥140	7279 (21)	114 (26)	551 (27)	2679 (25)	2857 (20)	1078 (17)		
Diastolic blood pressure (mmHg)	72 [65–80]	74 [65–81]	74 [65–80]	73 [65–80]	71 [65–80]	71 [65–80]	<.001	4
Mean arterial pressure (mmHg)	89 [81–98]	90 [80–100]	91 [82–100]	90 [82–100]	88 [80–97]	88 [80–97]	<.001	4
Heart rate (b.p.m.)	71 [62–81]	78 [68–90]	73 [65–84]	72 [63–82]	70 [62–80]	70 [61–80]	<.001	4
Heart rate (b.p.m.)^a^							<.001	4
50–70	15 925 (47)	139 (32)	818 (41)	4915 (46)	6838 (48)	3215 (50)		
<50	751 (2)	11 (2)	36 (2)	198 (2)	334 (2)	172 (3)		
>70	17 020 (51)	290 (66)	1125 (57)	5644 (52)	6943 (49)	3018 (47)		
EQ-5D-VAS	70 [50–80]	70 [50–80]	65 [50–80]	70 [50–80]	70 [50–80]	70 [50–80]	.028	45
EQ-5D-VAS							.035	45
0–25	868 (4)	<10	43 (5)	276 (5)	370 (4)	170 (4)		
26–50	4710 (24)	38 (27)	235 (29)	1474 (25)	1964 (24)	999 (24)		
51–75	8009 (41)	51 (36)	312 (38)	2389 (41)	3476 (42)	1781 (42)		
76–100	5780 (30)	44 (31)	230 (28)	1758 (30)	2448 (30)	1300 (31)		
Laboratory characteristics								
eGFR (mL/min/1.73 m^2^)	69 [52–88]	65 [43–90]	59 [38–81]	67 [48–87]	70 [54–88]	73 [57–89]	<.001	4
eGFR (mL/min/1.73 m^2^)^a^							<.001	4
≥60	21 616 (64)	254 (57)	961 (48)	6433 (60)	9403 (66)	4565 (70)		
30–59	10 749 (32)	131 (30)	718 (36)	3591 (33)	4454 (31)	1855 (28)		
<30	1611 (5)	58 (13)	318 (16)	749 (7)	388 (3)	98 (2)		
Potassium (mmol/L)	4.3 [4.0–4.5]	4.2 [3.9–4.4]	4.2 [3.9–4.5]	4.2 [4.0–4.5]	4.3 [4.0–4.6]	4.3 [4.0–4.6]	<.001	4
Potassium (mmol/L)^a^							<.001	4
3.5–5 (normokalaemia)	31 616 (94)	395 (90)	1841 (93)	10 049 (94)	13 196 (93)	6135 (95)		
<3.5 (hypokalaemia)	867 (3)	25 (6)	76 (4)	281 (3)	354 (2)	131 (2)		
>5 (hyperkalaemia)	1317 (4)	18 (4)	73 (4)	374 (3)	631 (4)	221 (3)		
Haemoglobin (g/L)	137 [124–149]	130 [116–145]	130 [116–143]	135 [121–147]	137 [125–148]	142 [130–154]	<.001	13
NT-proBNP (pg/mL)	2010 [813–4744]	3220 [1178–8006]	2803 [1077–7905]	2364 [956–5600]	1925 [798–4428]	1548 [648–3590]	<.001	19
NT-proBNP (pg/mL)^a^							<.001	19
1st tertile	9457 (33)	91 (24)	418 (26)	2505 (29)	4113 (34)	2330 (40)		
2nd tertile	9457 (33)	102 (27)	473 (29)	2764 (32)	4112 (34)	2006 (34)		
3rd tertile	9744 (34)	179 (48)	718 (45)	3390 (39)	3914 (32)	1543 (26)		
Comorbidities								
Smoking^a^	2978 (11)	52 (20)	135 (9)	952 (11)	1262 (11)	577 (11)	<.001	23
Diabetes^a^	7780 (23)	98 (22)	472 (24)	2302 (21)	3246 (23)	1662 (25)	<.001	3
Diabetes type							<.001	3
No	26 454 (77)	352 (78)	1533 (77)	8503 (79)	11 066 (77)	5000 (75)		
Type I	460 (1)	<10	32 (2)	172 (2)	206 (1)	43 (1)		
Type II	7298 (21)	91 (20)	438 (22)	2126 (20)	3027 (21)	1616 (24)		
Hypertension^a^	23 364 (66)	299 (63)	1495 (71)	7293 (65)	9873 (67)	4404 (65)	<.001	0
Ischaemic heart disease^a^	17 900 (51)	249 (53)	1156 (55)	5683 (51)	7668 (52)	3144 (47)	<.001	0
Myocardial infarction	13 280 (38)	201 (43)	868 (41)	4227 (38)	5661 (38)	2323 (35)	<.001	0
Revascularization	10 676 (31)	133 (30)	637 (32)	3278 (30)	4670 (32)	1958 (29)	<.001	2
Dilated cardiomyopathy	5217 (15)	72 (15)	181 (9)	1208 (11)	2646 (18)	1110 (16)	<.001	0
Peripheral artery disease^a^	3155 (9)	49 (10)	276 (13)	1021 (9)	1284 (9)	525 (8)	<.001	0
Atrial fibrillation/flutter^a^	18 921 (54)	214 (45)	1164 (56)	6173 (55)	7971 (54)	3399 (51)	<.001	0
Stroke^a^	4505 (13)	91 (19)	372 (18)	1484 (13)	1779 (12)	779 (12)	<.001	0
TIA	1021 (3)	12 (3)	71 (3)	371 (3)	396 (3)	171 (3)	.007	0
Anaemia^a^	8753 (29)	182 (43)	779 (42)	3153 (32)	3522 (28)	1117 (19)	<.001	13
Valvular disease^a^	6142 (17)	101 (21)	454 (22)	1902 (17)	2616 (18)	1069 (16)	<.001	0
Hyperkalaemia	735 (2)	14 (3)	67 (3)	274 (2)	284 (2)	96 (1)	<.001	0
Dialysis	372 (1)	15 (3)	63 (3)	159 (1)	103 (1)	32 (0)	<.001	0
Liver disease	900 (3)	36 (8)	42 (2)	253 (2)	406 (3)	163 (2)	<.001	0
COPD^a^	3950 (11)	55 (12)	269 (13)	1357 (12)	1640 (11)	629 (9)	<.001	0
Malignant cancer within 3 years^a^	4460 (13)	75 (16)	365 (17)	1565 (14)	1713 (12)	742 (11)	<.001	0
Musculoskeletal/connective tissue diseases within 3 years^a^	10 263 (29)	153 (32)	752 (36)	3481 (31)	4118 (28)	1759 (26)	<.001	0
Alcohol abuse^a^	1424 (4)	33 (7)	67 (3)	415 (4)	598 (4)	311 (5)	<.001	0
Charlson Comorbidity Index	2 [1–4]	3 [2–5]	3 [2–5]	3 [1–4]	2 [1–4]	2 [1–4]	<.001	0
Charlson Comorbidity Index							<.001	0
1–3	23 786 (68)	261 (55)	1113 (53)	7232 (65)	10 238 (70)	4942 (73)		
4–7	9985 (28)	168 (36)	826 (39)	3441 (31)	3971 (27)	1579 (23)		
≥8	1444 (4)	42 (9)	154 (7)	532 (5)	509 (3)	207 (3)		
Treatments								
ACEi/ARB/ARNi	32 619 (93)		966 (46)	10 376 (93)	14 549 (99)	6728 (100)	<.001	0
ACEi	16 406 (47)		520 (25)	5901 (53)	7423 (50)	2562 (38)	<.001	0
ARB	11 766 (33)		396 (19)	4051 (36)	5263 (36)	2056 (31)	<.001	0
ARNi	9332 (27)		130 (6)	1468 (13)	4315 (29)	3419 (51)	<.001	0
Beta-blocker	32 413 (92)		969 (46)	10 273 (92)	14 443 (98)	6728 (100)	<.001	0
MRA	21 109 (60)		132 (6)	1445 (13)	12 804 (87)	6728 (100)	<.001	0
SGLT2i	9428 (27)		26 (1)	316 (3)	2358 (16)	6728 (100)	<.001	0
Ivabradine	240 (1)	<10	17 (1)	60 (1)	110 (1)	47 (1)	.113	0
Loop diuretics	21 596 (61)	149 (32)	1260 (60)	7023 (63)	9320 (63)	3844 (57)	<.001	0
Digoxin	3662 (10)	22 (5)	166 (8)	1154 (10)	1684 (11)	636 (9)	<.001	0
Nitrate	6725 (19)	41 (9)	385 (18)	2313 (21)	2868 (19)	1118 (17)	<.001	0
Number of other CV and non-CV medications^a^							<.001	0
0–1	2159 (6)	166 (35)	140 (7)	651 (6)	793 (5)	409 (6)		
2–4	12 139 (34)	149 (32)	590 (28)	3675 (33)	5147 (35)	2578 (38)		
5–6	8773 (25)	58 (12)	500 (24)	2796 (25)	3747 (25)	1672 (25)		
7–8	6221 (18)	40 (8)	390 (19)	2058 (18)	2592 (18)	1141 (17)		
≥9	5923 (17)	58 (12)	473 (23)	2025 (18)	2439 (17)	928 (14)		
Device therapy^a^							<.001	0
No	29 319 (84)	396 (86)	1840 (89)	9768 (88)	11 794 (80)	5521 (82)		
CRT/ICD	5747 (16)	65 (14)	235 (11)	1368 (12)	2875 (20)	1204 (18)		

ACEi, angiotensin-converting enzyme inhibitor; ARB, angiotensin receptor blocker; ARNi, angiotensin receptor–neprilysin inhibitor; BMI, body mass index; COPD, chronic obstructive pulmonary disease; CRT, cardiac resynchronization therapy; CV, cardiovascular; EF, ejection fraction; eGFR, estimated glomerular filtration rate (calculated by the Chronic Kidney Disease Epidemiology Collaboration formula); EQ-5D-VAS, EuroQol 5-Dimension visual analogue scale; HF, heart failure; HHF, hospitalization for heart failure; ICD, implantable cardioverter-defibrillator; MRA, mineralocorticoid receptor antagonist; NT-proBNP, N-terminal pro-B-type natriuretic peptide; NYHA, New York Heart Association; RASi, renin–angiotensin system inhibitor; SGLT2i, sodium–glucose co-transporter 2 inhibitor; TIA, transient ischaemic attack.

^a^Included as covariates in multiple imputation models and multivariable logistic regression models and multivariable Cox proportional hazards regression models.

An explorative analysis was performed on the following outcomes: the composite of first HHF and cardiovascular death, first HHF, and cardiovascular death. Survival functions were depicted by cumulative incidence curves. Associations with outcomes of number of prescribed GDMT components, adherence (PDC ≥ vs <80%), and 1-year persistence (yes vs no) were assessed by crude and multivariable Cox regression models, with multivariable models adjusted for covariates reported in *Table 1*. Results were reported as hazard ratios (HR) with 95% CI. To mitigate potential temporal bias favouring quadruple therapy due to recent prognostic improvements in HFrEF and improved management of comorbidities,^16^ a sensitivity analysis restricted to patients enrolled 2021–23 was performed for the association between number of prescribed GDMT components and outcomes.

Missing data were handled by multiple imputation (10 sets, R-package *mice*^17^), including variables labelled in *Table 1* in the imputation model. Estimates and standard errors were pooled according to Rubin’s rules. No adjustments were performed for multiple comparisons due to the exploratory nature of the analysis. Two-sided *P* < .05 were considered statistically significant. The statistical software R 4.5.0 was used for all analyses. The R code for can be found https://github.com/KIHeartFailure/swedehf-hftreat.

## Results

### Patient characteristics

Of 35 215 patients with HFrEF enrolled in SwedeHF between 2016 and 2023, the median age was 74 (IQR 64–80) years, 28% were female, 57% were enrolled in 2016–20 (triple therapy era), and 43% in 2021–23 (quadruple therapy era). Approximately 42% had history of a HHF within the previous year, 38% had New York Heart Association (NYHA) class III/IV, the median N-terminal pro-B-type natriuretic peptide (NT-proBNP) was 2010 (IQR 813–4744) pg/mL, and 5% had an estimated glomerular filtration rate (eGFR) < 30 mL/min/1.73 m^2^.

Several patient characteristics differed according to number of prescribed GDMT components (*Table 1*). Those with more than one GDMT component were slightly younger [median (IQR) age 77 (69–85), 75 (66–82), 73 (64–79), and 72 (62–78) years with mono, double, triple, and quadruple therapy, respectively), had lower NYHA class and NT-proBNP levels, but also lower EF and Charlson Comorbidity Index, and were more often referred for follow-up in specialty care or in HF nurse clinic. Those without any GDMT at baseline represented a small (∼1%) and apparently distinct sub-group, being younger [median (IQR) age 73 (61–80) years] than those with mono or double therapy, with longer HF duration, more likely a recent HHF, higher NYHA classes, and higher NT-proBNP levels.

### Implementation of guideline-directed medical therapy over time

**Figure 1:**
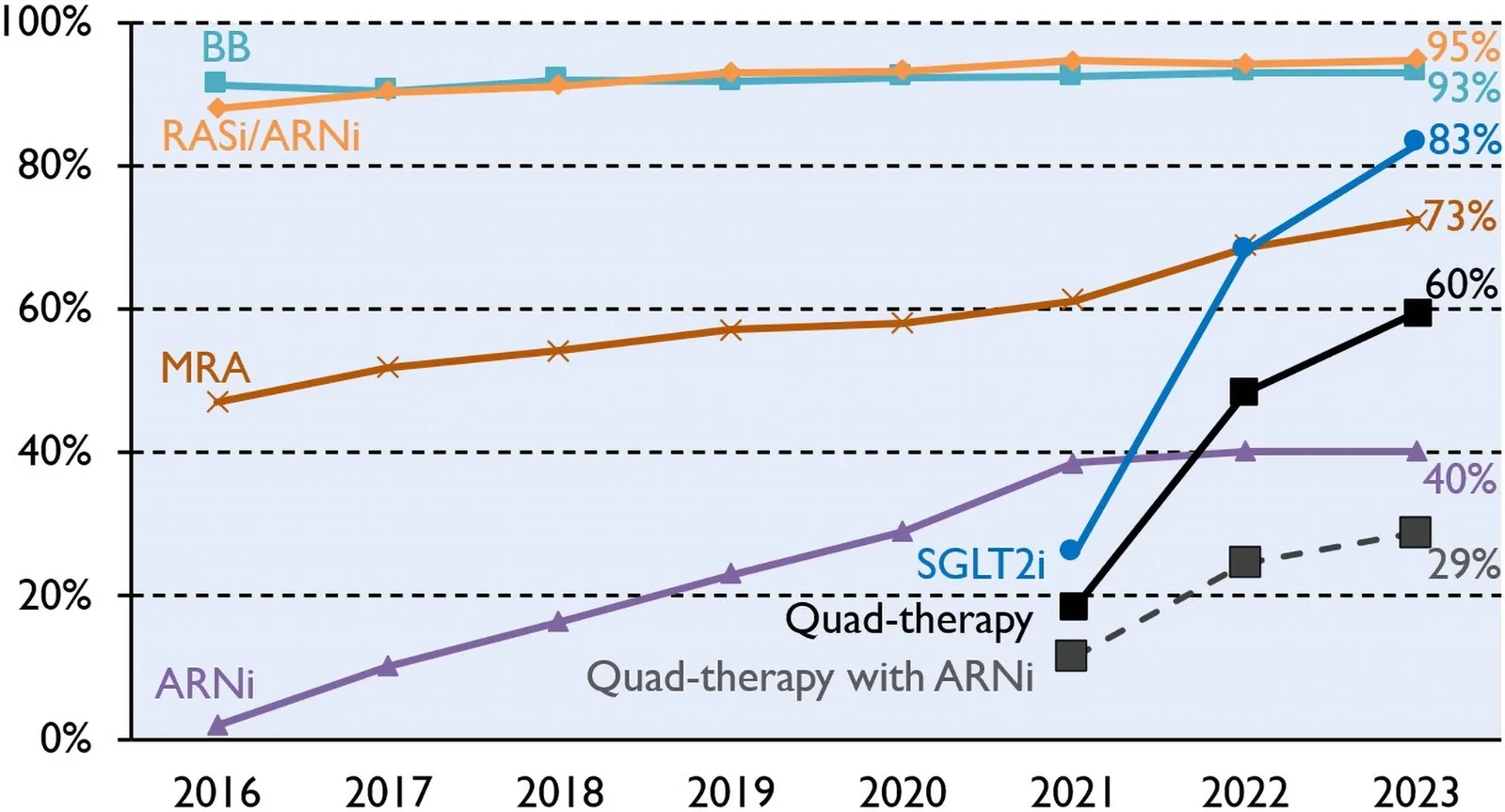
Implementation of guideline-directed medical therapy over time. ARNi, angiotensin receptor–neprilysin inhibitor; BB, beta-blocker; GDMT, guideline-directed medical therapy; MRA, mineralocorticoid receptor antagonist; RASi, renin–angiotensin system inhibitor; SGLT2i, sodium–glucose co-transporter 2 inhibitor; Quad, quadruple

Between 2016 and 2023, the proportion of patients prescribed HFrEF pharmacotherapy increased for all GDMT components. The use of SGLT2i and quadruple therapy increased rapidly after their introduction for HFrEF in 2021. As of 2023, approximately 93% used a beta-blocker, 95% RASi/ARNi, 40% ARNi, 73% MRA, 83% SGLT2i, 60% quadruple therapy, and 29% quadruple therapy with ARNi (*Figure 1*).

For beta-blockers, RASi/ARNi, and MRA, a minority of patients were prescribed at least 100% of guideline-recommended target dose. The proportion of patients receiving at least 100% of target doses increased over time for RASi/ARNi and MRA, but not for beta-blockers, and in 2023 reached 23% for beta-blockers, 36% for RASi/ARNi, 10% for MRA, and 82% for SGLT2i; in 2023, approximately 5.7% of patients were prescribed target doses for all four pillars of quadruple GDMT and 4.4% of all four pillars of quadruple GDMT including ARNi (*Table 2*).

**Table 2:** Proportion of all patients prescribed 100% of target dose^a^ per therapy over time

	2016	2017	2018	2019	2020	2021	2022	2023
Beta-blocker	24.5%	25.7%	26.8%	26.7%	26.6%	25.4%	25.6%	23.0%
RASi/ARNi	27.9%	32.9%	33.7%	36.3%	38.5%	37.5%	37.5%	36.3%
MRA	4.1%	6.4%	7.4%	8.0%	8.2%	8.5%	8.8%	9.8%
SGLT2i						25.3%	67.2%	82.2%
Quadruple therapy						2.1%	4.2%	5.7%
Quadruple therapy with ARNi						1.7%	3.2%	4.4%

ARNi, angiotensin receptor–neprilysin inhibitor; MRA, mineralocorticoid receptor antagonist; RASi, renin–angiotensin system inhibitor; SGLT2i, sodium–glucose co-transporter 2 inhibitor.

^a^Target dose per substance outlined in Supplementary data online, *Table S3*.

### Implementation of guideline-directed medical therapy across subgroups in the quadruple therapy era

**Figure 2:**
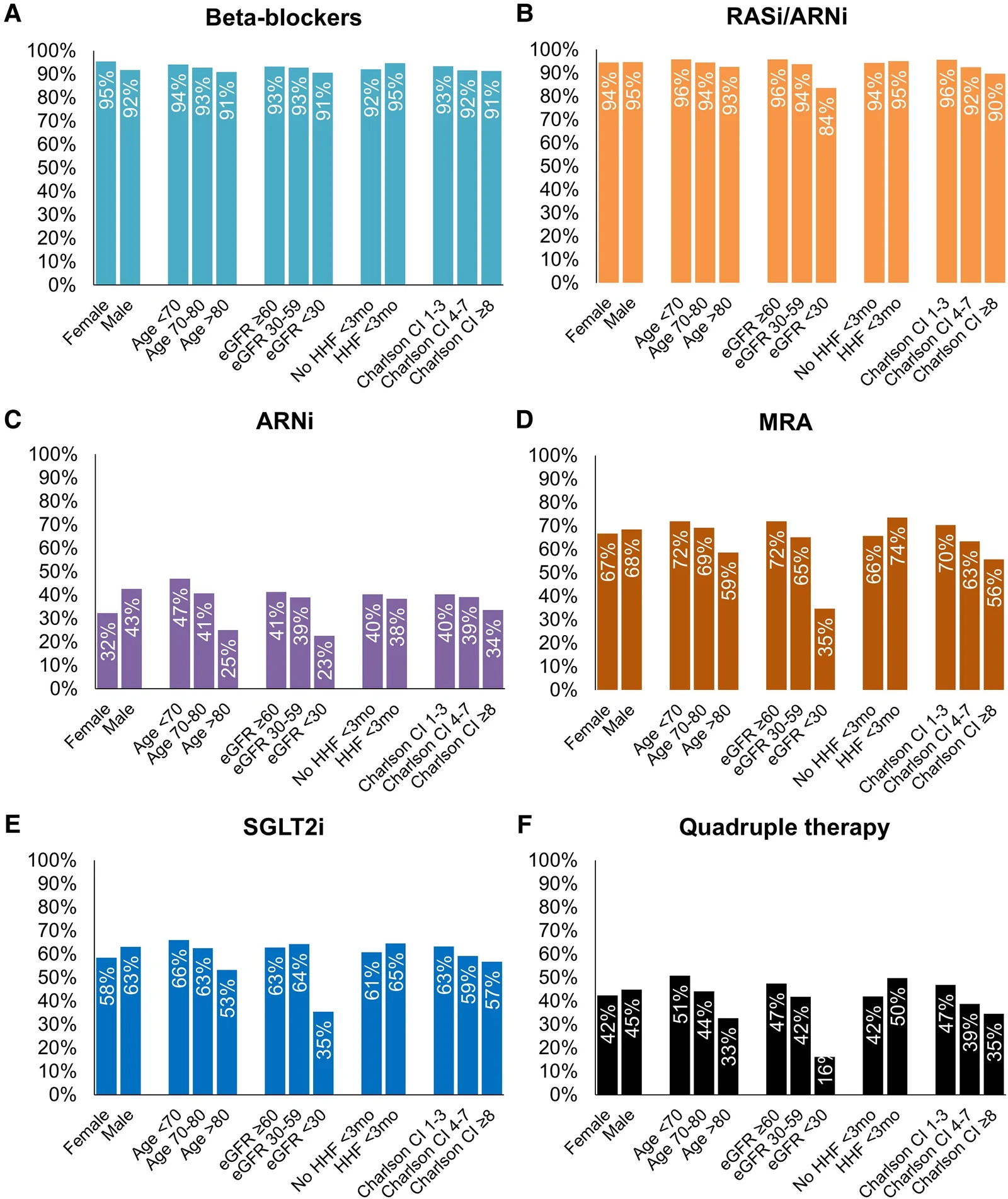
Implementation of guideline-directed medical therapy across subgroups in the period 2021–23 (quadruple therapy era). ARNi, angiotensin receptor–neprilysin inhibitor; CI, Comorbidity Index; eGFR, estimated glomerular filtration rate; HHF, hospitalization for heart failure; GDMT, guideline-directed medical therapy; MRA, mineralocorticoid receptor antagonist; RASi, renin–angiotensin system inhibitor; SGLT2i, sodium–glucose co-transporter 2 inhibitor; Quad, quadruple

In the period 2021–23, beta-blockers were slightly more used in females (95%) than males (92%), but females were less commonly prescribed with ARNi (32% vs 43%), SGLT2i (58% vs 63%), and quadruple therapy (42% vs 45%) (*Figure 2*). Guideline-directed medical therapy components, as well as quadruple therapy, were less commonly prescribed with increasing age, increasing Charlson Comorbidity Index, decreasing eGFR (except greater use of SGLT2i in the eGFR range 30–59 mL/min/1.73 m^2^), and in patients without vs with previous HHF in the past 3 months (except for ARNi).

### Patient adherence and persistence

**Figure 3:**
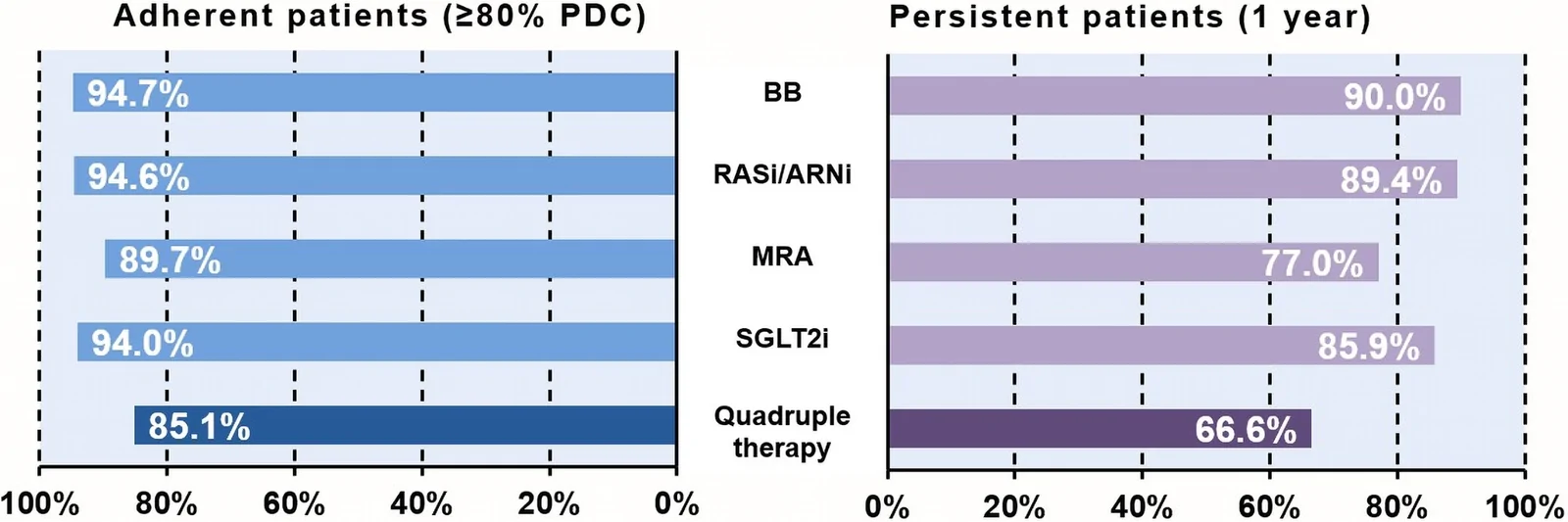
Proportion of patients with good adherence (proportion of days covered ≥80%) and 1-year persistence to heart failure with reduced ejection fraction guideline-directed medical therapy. ARNi, angiotensin receptor–neprilysin inhibitor; BB, beta-blocker; GDMT, guideline-directed medical therapy; HFrEF, heart failure with reduced ejection fraction; MRA, mineralocorticoid receptor antagonist; RASi, renin–angiotensin system inhibitor; SGLT2i, sodium–glucose co-transporter 2 inhibitor; PDC, proportion of days covered; Quad, quadruple

Overall high patient adherence was observed for all GDMT components (*Figure 3*). The proportion of patients with good adherence (≥80% PDC) was 95% for beta-blockers, 95% for RASi/ARNi, 90% for MRA, 94% for SGLT2i, and 85% for quadruple therapy.

The proportion of patients persisting to prescribed therapy at 0.5, 1, 1.5, and 2 years, respectively, was 92%, 90%, 89%, and 88% for beta-blockers; 92%, 89%, 87%, and 87% for RASi/ARNi; 82%, 77%, 75%, and 72% for MRA; and 90%, 86%, 85%, and 85% for SGLT2i. Approximately 67% persisted on quadruple therapy at 1 year. Regarding RASi and ARNi, ARNi displayed the best adherence and 1-year persistence (93% and 88%) followed by angiotensin receptor blockers (82% and 66%) and with angiotensin-converting enzyme inhibitors last (79% and 60%). Patients with vs without a HHF at the time of SwedeHF registration or in the past 3 months were overall similarly likely to have good adherence and 1-year persistence (see Supplementary data online, *Table S5*).

### Patient characteristics associated with adherence and persistence

Male sex was independently associated with lower adherence to beta-blockers and RASi/ARNi, and older age with better adherence to RASi/ARNi and SGLT2i. Among socioeconomic variables, living alone was associated with lower adherence to beta-blockers and RASi/ARNi and higher income with better adherence to all GDMT components. Several parameters and comorbidities potentially linked with tolerability issues were observed as independent predictors of lower adherence, including low blood pressure (RASi/ARNi), low heart rate (beta-blockers), lower eGFR (RASi/ARNi, MRA, and SGLT2i), and hyperkalaemia (MRA). Variables linked with poor health habits were associated with worse adherence, e.g. alcohol abuse (beta-blockers, RASi/ARNi, and MRA) and smoking (beta-blockers and MRA). A greater number of co-prescribed medications were associated with better adherence to beta-blockers and RASi/ARNi.

Predictors of 1-year persistence were overall directionally consistent with those of patient adherence. A full list of all independent predictors of patient adherence and 1-year persistence are detailed in Supplementary data online, *Tables S6* and *S7*.

### Explorative associations between prescriptions, patient adherence, persistence, and outcomes

**Figure 4:**
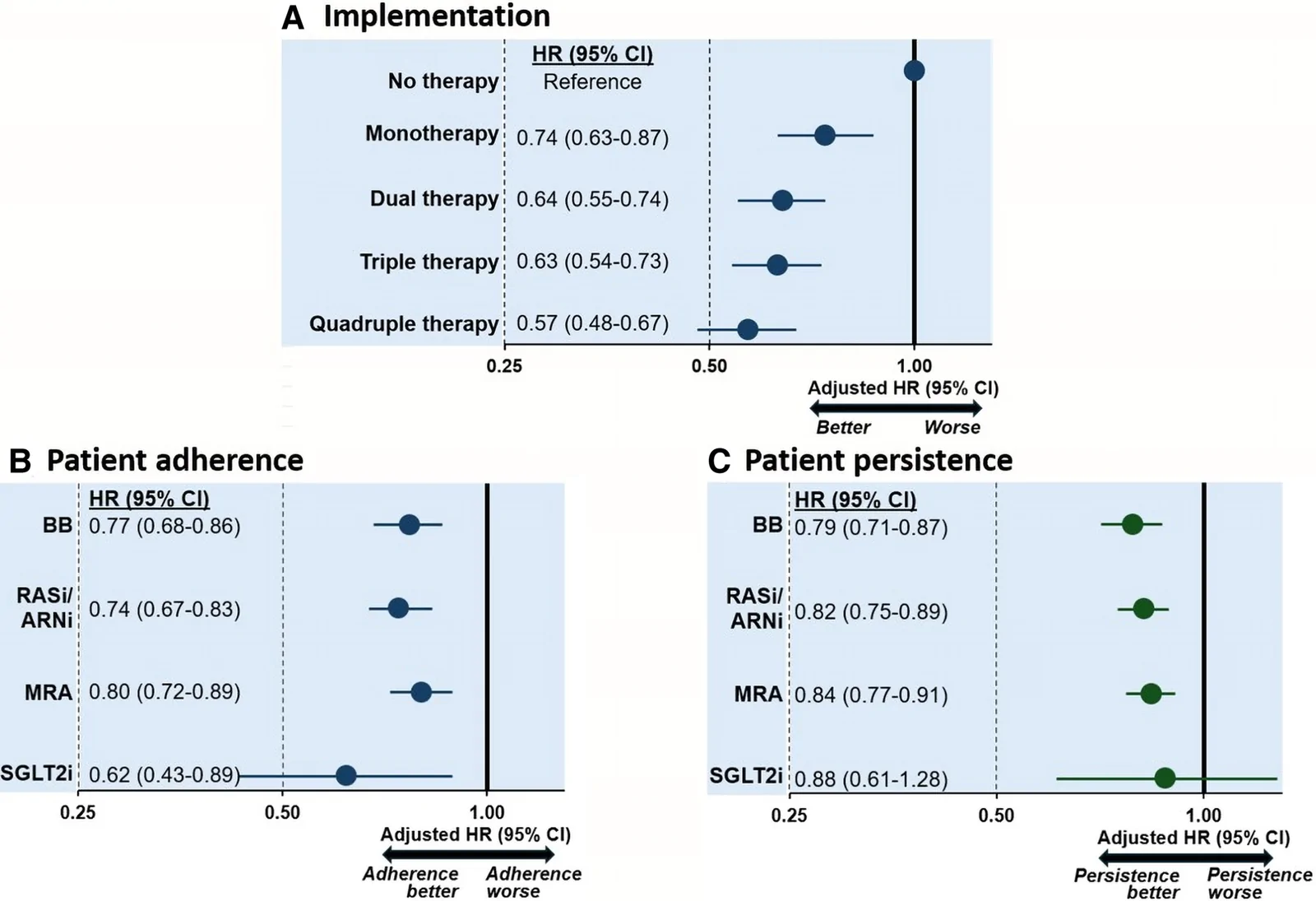
Associations between (*A*) implementation of, (*B*) good patient adherence (proportion of days covered ≥80%) to, and (*C*) 1-year patient persistence to failure with reduced ejection fraction guideline-directed medical therapy and hospitalization for heart failure/cardiovascular death. ARNi, angiotensin receptor–neprilysin inhibitor; BB, beta-blocker; CI, confidence interval; GDMT, guideline-directed medical therapy; HFrEF, heart failure with reduced ejection fraction; HHF, hospitalization for heart failure; HR, hazard ratio; MRA, mineralocorticoid receptor antagonist; PDC, proportion of days covered; RASi, renin–angiotensin system inhibitor; SGLT2i, sodium–glucose co-transporter 2 inhibitor

Over a median follow-up of 2.3 years (interquartile range: 0.9–3.0) years, the incidence rate of experiencing a HHF/cardiovascular death was 16 (95% CI 16–16) per 100 patient-years [14 (95% CI 12–13) for HHF and 7 (95% CI 6–7) for cardiovascular deaths). As compared with having no prescribed GDMT at index, an increasing number of prescribed GDMTs were associated with progressively lower risk for HHF/cardiovascular death in crude and adjusted analyses, e.g. monotherapy [crude hazard ratio (HR) 0.81 (95% CI 0.55–1.19); adjusted HR 0.64 (0.42–0.95)], dual therapy [crude HR 0.64 (0.45–0.91); adjusted HR 0.61 (0.42–0.89)], triple therapy [crude HR 0.56 (0.40–0.79); adjusted HR 0.58 (0.40–0.83)], and quadruple therapy [crude HR 0.48 (0.34–0.68); adjusted HR 0.54 (0.37–0.78)] (*Figure 4*).

In crude and adjusted analyses, having good adherence during the first year post-index was associated with lower risk for HHF/cardiovascular death for beta-blockers [crude HR 0.70 (0.62–0.79); adjusted HR 0.77 (0.68–0.86)], RASi/ARNi [crude HR 0.54 (0.48–0.60); adjusted HR 0.74 (0.67–0.83)], MRA [crude HR 0.61 (0.55–0.67); adjusted HR 0.80 (0.72–0.89)], and SGLT2i [crude HR 0.52 (0.37–0.74); adjusted HR 0.62 (0.43–0.89)].

In crude and adjusted analyses, persisting use of therapy at 1 year post-index was associated with lower risk for HHF/cardiovascular death for beta-blockers [crude HR 0.81 (0.74–0.89); adjusted HR 0.79 (0.71–0.87)], RASi/ARNi [crude HR 0.65 (0.60–0.71); adjusted HR 0.82 (0.75–0.89)], and MRA [crude HR 0.67 (0.61–0.73); adjusted HR 0.84 (0.77–0.91)], but not significantly for SGLT2i [crude HR 0.76 (0.53–1.09); adjusted HR 0.88 (0.61–1.28)].

### Sensitivity analyses

The adjusted association between number of prescribed GDMT components and HHF/cardiovascular death was consistent with the main analysis when restricted to those enrolled 2021–23 (see Supplementary data online, *Table S8*). The proportions of patients with good adherence (≥80% PDC) and 1-year persistence, respectively, assessed separately among *de novo* users (beta-blockers: 95% and 89%; RASi/ARNI: 95% and 89%, MRA: 90% and 75%, SGLT2i: 94% and 85%) were consistent with those observed in the main analysis including also prevalent users. When defining good adherence as ≥88% PDC, the proportion of patients with good adherence was slightly lower than in the main analysis (beta-blockers: 92%; RASi/ARNI: 92%, MRA: 87%, SGLT2i: 92%, quadruple therapy: 80%).

## Discussion

Despite therapy uptake being increasingly recognized as an unmet need in HFrEF, comprehensive assessments spanning from physician prescriptions to patient adherence in the era of quadruple pharmacotherapy have been limited. In this contemporary nationwide cohort of >35 000 patients with HFrEF, we observed overall high GDMT uptake in terms of prescriptions and patient adherence. The proportion of patients receiving quadruple therapy increased rapidly since 2021 (time of ESC HF Guidelines), reaching ∼60% in 2023. The proportion of patients with good adherence was ∼90% or higher for all drug classes and ∼85% for quadruple therapy overall. As of 2023, use of SGLT2i overtook MRA, which remains the least prescribed (73%), least adhered to (90%), and least persisted (77% at 1 year) among the GDMTs. In an explorative analysis, patient adherence and 1-year persistence were associated with lower risk for HHF or cardiovascular death after adjustments for measured confounders (*Structured Graphical Abstract*). Our findings exemplify that high GDMT penetrance is achievable in an HFrEF cohort predominantly reflecting specialized cardiology care in a universal healthcare system, but that even in this setting there persist major areas of improvement, including suboptimal attainment of target doses and frequent discontinuation of prescribed therapies. Addressing patient adherence and persistence to GDMT might further improve outcomes.

Underuse of HFrEF GDMT has been shown to be a major challenge in the era of triple therapy across all studied healthcare settings,^5^ but with limited data available since the addition of SGLT2i as a fourth pillar of HFrEF pharmacotherapy in 2021.^1,18^ Previous data from SwedeHF showed that virtually no improvements were made in the implementation of HFrEF therapies between 2003 and 2012 and that this lack of progress was coupled with stable mortality rates.^19^ Since then, as our data show, all evidence-based pharmacotherapies for HFrEF have seen increasing implementation, although target doses of neurohormonal therapies were still achieved in a minority of patients. This progress mirrors recently reported prognostic improvements for patients with HFrEF in Sweden.^16^

What are the potential reasons for the observed positive shift in HFrEF therapy implementation in Sweden? First, it is plausible that increased attention to extensive underuse of therapies has contributed to improved awareness among healthcare providers. Second, the widespread integration of the HF-specific national registry (SwedeHF) into cardiology clinics has likely heightened attention to treatment gaps at both national and local levels, aligning with the principle that measurement drives improvement. Third, the increased adoption of HF nurse-led clinics in Sweden enables tighter follow-up during up-titration or vulnerable phases that carry risk for treatment de-escalation. Fourth, recent years’ novel therapies might act as ‘enablers’ to other HFrEF therapies. For example, in PARADIGM-HF and EMPEROR-Reduced, respectively, ARNi and SGLT2i were linked with greater use of MRA.^9,10^ Indeed, in our data, the implementation of MRA improved year-by-year throughout the 2016–23 period, but with a steepening slope following the adoption of SGLT2i for HFrEF therapy in 2021. Fifth, past emphasis on sequential addition and uptitration of one GDMT followed by the next may have caused delays, whereas the 2021 Guideline recommendation of simultaneous or near simultaneous introduction of all four may have had considerable effects on all aspects of GDMT uptake. Furthermore, following STRONG-HF,^20^ the 2023 focused update of the ESC HF guidelines strengthened the emphasis on rapid treatment intensification pre- and post-discharge among patients hospitalized for HF.^21^ Finally, our cohort consisted of patients with HFrEF enrolled in a quality registry predominantly reflecting secondary/tertiary care, which might also partly explain the observation of better GDMT implementation than in studies using electronic health records from broader and less selected populations, but with less data granularity for strictly identifying indications (e.g. EF) for drugs.^6^ While our study is performed in a setting where GDMT implementation is more likely achievable, it should be underscored that a large proportion of patients (∼40%) did not receive quadruple therapy at the end of the study period, and receiving four GDMT components at target doses remained rare (∼6%). Although some patients might have characteristics that truly prevent the achievement of full GDMT, our findings further highlight that the implementation of guidelines in the real world is a complex process, with barriers at several levels, i.e. physician-related, patient-related, therapy-related, and healthcare system-related, which are difficult to address even in well-structured universal healthcare system.

Patient adherence and persistence to therapy are well-known challenges in chronic cardiovascular conditions and core determinants of real-world treatment uptake.^3,22^ In a recent cardiologist survey from the Heart Failure Association of the ESC, 42% of participants referred to patient adherence as a major clinical barrier to implementation of GDMT in HFrEF.^23^ However, comprehensive assessments of patient adherence to HFrEF therapy in the era of quadruple therapy have been lacking. Our data suggest that patient adherence to pharmacotherapy in HFrEF (90%–95% of patients having good adherence across drug classes) is overall higher than in other chronic cardiovascular settings such as hypertension and hyperlipidaemia, possibly due to HF being more likely symptomatic and recognized by the patient as a serious condition. Our adherence estimates are comparable to reports from Norway (proportion with good adherence: beta-blockers 83%, RASi/ARNi 84%–91%, MRA 61%),^24^ a universal healthcare system with similarities to Sweden, but appear considerably higher than in the USA (beta-blocker 61%, RASi/ARNi 35%–59%, MRA 36%).^25,26^ We believe that two main attributes of the our study setting might contribute to higher adherence than might be observed in other contexts. First, high co-payments have been associated with lower adherence in HF,^27^ and in Sweden, medication costs are covered once an annual out-of-pocket spend threshold (in 2023 ∼230€) has been reached. Second, the SwedeHF setting has relatively high penetrance of follow-up in HF nurse-led clinics, enabling more frequent out-patient contacts which has also been linked with better patient adherence in HF.^28^ Despite the relatively high adherence observed in our study, there is still room for improvement particularly for the long-term persistence to quadruple therapy which was only ∼67% at 1 year, mainly limited by lower persistence to MRA (77%).

In an explorative outcome analysis, patient adherence to all GDMTs and 1-year persistence to all GDMTs except for SGLT2i were associated with lower risk for HHF/cardiovascular death before and after adjustments for measured confounders. For SGLT2i, although the point estimate was consistent with what was observed for the other drugs, the association between 1-year persistence and HHF/cardiovascular death was not statistically significant, likely due to the limited sample size. It is important to note that the observational nature of this analysis precludes causal conclusions; even after extensive adjustments, unmeasured confounders might partly explain our findings. In the CHARM trial, poor adherence even to placebo was associated with adverse prognosis, highlighting that prognostic associations of poor therapy adherence might not only be explained by diluted therapeutic benefits but also by several potential confounders, such as overall worse health behaviour, disease severity, and comorbid condition predisposing to tolerability issues.^29^

A first step in addressing patient adherence is to recognize when there is risk for non-adherence. We identified three key clusters of patient characteristics that appeared broadly and independently linked with greater risk for non-adherence to HFrEF GDMT: (i) characteristics linked with tolerability issues, e.g. hypotension (RASi/ARNi), bradycardia (beta-blockers), and hyperkalaemia (MRA); (ii) factors linked with overall poor health behaviour (e.g. smoking, alcohol abuse); and (iii) poor socioeconomic status. Improved digital health infrastructures and the integration of HF registries in the clinical workflow might facilitate identification of—and possibly screening for—gaps in treatment uptake, from implementation to patient adherence and persistence. Although meta-analyses suggest that medication adherence interventions in HF improve clinical outcomes,^30^ HF-specific evidence on how to best improve medication adherence is limited. As highlighted in a scientific statement from the Heart Failure Association of the ESC,^3^ potential strategies to improve patient adherence might include nudges such as text message reminders,^31,32^ patient education, integration of HF nurses and pharmacists in patient follow-up,^33^ and medication regimen rationalization. Single pill combinations have been shown to improve adherence as well as outcomes in other cardiovascular diseases and are being studied in HFrEF (NCT04633005, NCT06029712).^34^

### Strengths and limitations

The primary strength of this study was the use of a large, contemporary, and well-characterized HF cohort, with virtually complete access to drug prescriptions, and virtually complete follow-up for cause-specific outcomes. The ability to ascertain prescribed doses from free text dose instructions represents a strength in relation to several prior studies of patient adherence, which have relied on the time interval between initial pharmacy refills to infer the intended dose. There are also key limitations that need to be acknowledged when interpreting our results. First, the Swedish healthcare system, and the SwedeHF setting in particular, has several attributes that might positively affect GDMT uptake, making generalizability to other healthcare systems uncertain. Moreover, only a subset of the Swedish HF population is captured in SwedeHF, where patients are predominantly seen in specialty care, are less likely female, receive better treatment, and have better prognosis.^35^ Therefore, it is likely that the treatment uptake observed in this study approaches a ‘best case scenario’, reflecting the secondary/tertiary care environment of a country with strategies to address barriers to GDMT implementation are in place. Our results may not be generalizable to other healthcare systems and less specialized or primary care settings, where lower uptake both in terms of implementation and patient adherence might be expected. Second, although standard methods were used to estimate adherence via pharmacy refills, these methods involve a multitude of investigator degrees of freedom and have inherent limitations, including that retrieved medications are not necessarily consumed and inability to fully account for doses consumed during hospital stays. Third, we lacked data on clinical parameters (potassium, blood pressure, heart rate) during follow-up and could not distinguish between non-adherence/non-persistence caused by patient preferences, non-compliance, and clinically motivated reasons such as side effects or palliation. Fourth, given the aim of providing a general picture of treatment uptake, this study included prevalent users, who are expected to have higher adherence/persistence than first-time users since early discontinuations due to uncovered side effects are less likely, although a sensitivity analysis in *de novo* users showed overall consistent results. Fifth, implementation was defined according to prescriptions dispensed in pharmacies, meaning that those who never dispensed their prescription were considered as non-users. This might contribute to a slight underestimation of therapy implementation and a slight overestimation of patient adherence. Finally, the observational design precludes causal conclusions, and our findings should therefore be interpreted as descriptive.

## Conclusion

As of 2023, use of quadruple HFrEF therapy reached ∼60% and was mostly limited by the lower adoption of MRA (∼73%). Patient adherence to HFrEF foundational therapies was high and appeared associated with better prognosis. These data suggest that improving the uptake of quadruple HFrEF pharmacotherapy is achievable in specific healthcare settings, although suboptimal dosing and frequent discontinuations remain important areas of further implementation, suggesting a need for sustained monitoring for preventable discontinuations. Efforts targeting physician-level implementation, healthcare system-level, and patient-level adherence and persistence are key to transport the effect in trials to the real-world HFrEF population.

## Supplementary Material

ehag199_Supplementary_Data

## Declarations

### Data Availability

Sharing of the data underlying this article may be considered on reasonable request to the corresponding author, provided that data sharing is permitted by European Union General Data Protection Regulation regulations and appropriate ethics committees, including the Swedish Ethical Review Authority.
