# Supplementary material for: Heart failure with reduced ejection fraction in Sweden: patient adherence and persistence to quadruple pharmacotherapy prescription
Source: Eur Heart J. 2026 Apr 1;47(32):4442–58. doi: 10.1093/eurheartj/ehag199 (PMC13500168; doi:10.1093/eurheartj/ehag199)
Supplement: Supplementary file 1 [file ehag199_supplementary_data.docx]

### SUPPLEMENT

___________________________________________________________________

**Heart failure with reduced ejection fraction in Sweden: patient adherence and persistence to quadruple pharmacotherapy prescription**

___________________________________________________________________

Table of contents

[Table S1. Overview of data sources.	3](#_Toc215534307)

[Table S2. Patient selection flowchart.	4](#_Toc215534308)

[Table S3. Evidence-based doses of disease-modifying drugs in key randomized trials in patients with HFrEF	5](#_Toc215534309)

[Table S4. Data sources and diagnosis/procedure codes for variable definitions.	6](#_Toc215534310)

[Table S5. Proportion of patients with good adherence and 1-year persistence according to recent HF hospitalization status.	8](#_Toc215534311)

[Table S6. Independent predictors of patient adherence.	9](#_Toc215534312)

[Table S7. Independent predictors of 1-year patient persistence.	11](#_Toc215534313)

[Table S8. Crude and adjusted associations between number of GDMT components and time to the composite of first HF hospitalization and cardiovascular death.	13](#_Toc215534314)

[References	14](#_Toc215534315)

### Table S1. Overview of data sources.

| **Register name** | **Administrator** | **Type of data** | **Start year** |
| --- | --- | --- | --- |
| Register of the Total Population^111^ | Statistics Sweden | Data on all persons registered in Sweden, including birth date, sex, place of residence (parish, municipality, or county, depending on availability of reference individuals), last immigration and emigration dates, and identity of parents/siblings | 1968- |
| Longitudinal Integrated database for health insurance and labour market studies^2^ | Statistics Sweden | Type of work, compensations from employment, entrepreneurial activities, studies, national military service, illness, parental leave, unemployment, labour market activity, rehabilitation, partial retirement, early retirement, retirement, occupational pension, annuities, social assistance, private pensions, country of birth and parental countries of birth, place of employment, highest level of education | 1990- |
| National Patient Register^3^ | Swedish National Board of Health and Welfare | Data from outpatient visits and inpatient care in hospital-based care, including date of visit, primary and secondary diagnoses and procedures. Primary care is not included. | 1964- Hospitalizations  1987- Nationwide coverage  1997- Outpatient surgery  2001- Specialized outpatient visits |
| Cause of Death Register^4^ | Swedish National Board of Health and Welfare | Date and underlying as well as contributing cause of death. | 1961- |
| National Prescribed Drug Register^5^ | Swedish National Board of Health and Welfare | Names and Anatomical Therapeutic Chemical Classification of prescribed drugs dispensed from pharmacies. The coverage of the register is complete for prescriptions in ambulatory care (both specialist and primary care), while in-hospital drug treatment is not recorded. | July 2005- |
| Swedish Heart Failure Registry^6^ | Uppsala Clinical Research Center | The inclusion criterion is clinician-judged heart failure, since 2017 defined by International Statistical Classification of Diseases, 10th Revision I50.0, I50.1, I50.9, I42.0, I42.6, I42.7, I25.5, I11.0, I13.0, I13.2 | 2000 (2003 on a national level)- |

### Table S2. Patient selection flowchart.

| **Inclusion/exclusion criteria** | **N** |
| --- | --- |
| No of posts received | 246,769 |
| Exclude posts with duplicated index dates | 246,146 |
| Exclude posts with index date < 2000-01-01 | 246,143 |
| Exclude posts with index date > 2023-12-31 | 243,917 |
| Exclude posts < 18 years | 243,900 |
| Exclude posts that are not present in Statistics Sweden’s register | 243,489 |
| Exclude posts with reused personal identification numbers | 243,224 |
| Exclude posts with end of follow-up < index | 243,213 |
| Exclude posts with end of follow-up ≤ index date (died in hospital) | 239,931 |
| Exclude posts with missing ejection fraction | 195,584 |
| Include posts with ejection fraction < 40% | 104,417 |
| Include posts with index year ≥ 2016 | 47,167 |
| Include the last post / patient | 35,215 |

### Table S3. Evidence-based doses of disease-modifying drugs in key randomized trials in patients with HFrEF

**ACEi/ARB**

Captopril, 50 mg t.i.d. (150 mg / day)

Enalapril, 10 mg b.i.d. (20 mg / day)

Lisinopril, 20 mg o.d. (20 mg / day)

Ramipril, 5 mg b.i.d. (10 mg / day)

Trandolapril, 4 mg o.d. (4 mg / day)

Candesartan, 32 mg o.d. (32 mg / day)

Losartan, 150 mg o.d. (150 mg / day)

Valsartan, 160 mg b.i.d. (320 mg / day)

**ARNi**

Sacubitril/valsartan, 97/103 mg b.i.d. (194/206 mg / day)

**Beta-blockers**

Bisoprolol, 10 mg o.d. (10 mg / day)

Carvedilol, 50 mg b.i.d. (100 mg / day)

Metoprolol, 200 mg o.d. (200 mg / day)

Nebivolol, 10 mg o.d. (10 mg / day)

**MRA**

Eplerenone, 50 mg o.d. (50 mg / day)

Spironolactone, 50 mg o.d. (50 mg / day)

**SGLT2i**

Dapagliflozin, 10 mg o.d. (10 mg / day)

Empagliflozin, 10 mg o.d. (10 mg / day)

**Abbreviations:** ACEi, angiotensin-converting enzyme inhibitor; ARNi, angiotensin receptor–neprilysin inhibitor; ARB, angiotensin receptor blocker; HFrEF, heart failure with reduced ejection fraction; MRA, mineralocorticoid receptor antagonist; SGLT2i, sodium–glucose cotransporter 2 inhibitor

### Table S4. Data sources and diagnosis/procedure codes for variable definitions.

| **Variable** | **Data source/definition** |
| --- | --- |
| Index year | SwedeHF |
| Sex | SwedeHF |
| Age | SwedeHF |
| Duration of HF | SwedeHF |
| Previous HFH within 3 months | NPR: ICD-10 I110, I130, I132, I255, I420, I423, I425-9, I43, I50, J81, K761, R570 ^d^ |
| Previous HFH within 1 year | NPR: ICD-10 I110, I130, I132, I255, I420, I423, I425-9, I43, I50, J81, K761, R570 ^d^ |
| Follow-up referral HF nurse clinic | SwedeHF |
| Follow-up referral speciality | SwedeHF |
| LVEF | SwedeHF |
| NYHA class | SwedeHF |
| BMI | SwedeHF |
| Systolic blood pressure | SwedeHF |
| Diastolic blood pressure | SwedeHF |
| Mean arterial pressure | SwedeHF |
| Heart rate | SwedeHF |
| eGFR | SwedeHF CKD-EPI 2021^7^ |
| Potassium | SwedeHF |
| Hemoglobin | SwedeHF |
| NT-proBNP | SwedeHF |
| Smoking | SwedeHF |
| Diabetes | SwedeHF |
| Diabetes type | SwedeHF |
| Hypertension | SwedeHF; NPR: ICD-10 I10-5 ^b^ |
| Ischemic heart disease | SwedeHF; NPR: ICD-9/10 410-4, I20-5, Z951, Z955 Procedure FNG, FNA, FNB, FNC, FND, FNE, FNF, FNH ^b^ |
| Myocardial infarction | NPR: ICD-9/10 410, 412, I21, I22, I252 ^b^ |
| Revascularization | SwedeHF |
| Dilated cardiomyopathy | NPR: ICD-10 I420 ^b^ |
| Peripheral artery disease | NPR: ICD-10 I70-3 ^b^ |
| Atrial fibrillation/flutter | SwedeHF; NPR: ICD-10 I48 ^b^ |
| Stroke | NPR: ICD-9/10 430-4, 438, I60-4, I690-4 ^b^ |
| TIA | NPR: ICD-10 G45 ^b^ |
| Anemia | SwedeHF Female Hb < 120 g/L/Male Hb < 130 g/L |
| Valvular disease | NPR: ICD-10 I05-8, I34-9, Q22, Q230-3, Q230-3, Q235-9, Z952-4 ^b^ |
| Hyperkalemia | NPR: ICD-10 E875 ^b^ |
| Dialysis | NPR: ICD-10 Z491, Z492 Procedure DR014, DR015, DR016, DR020, DR012, DR013, DR023, DR024, TJA33, TJA35 ^b^ |
| Liver disease | NPR: ICD-10 B18, I85, I864, I982, K70, K710, K711, K713-7, K72-4, K760, K762-9 ^b^ |
| COPD | NPR: ICD-10 J40-4 ^b^ |
| Malignant cancer within 3 years | NPR: ICD-10 C ^b^ |
| Muscoloskeletal/connective tissue diseases within 3 years | NPR: ICD-10 M ^b^ |
| Alcohol abuse | NPR: ICD-10 E244, E52, F10, G312, G621, G721, I426, K292, K70, K860, O354, P043, Q860, T51, Z502, Z714 Cause Y90, Y91 ^b^ |
| Charlson Comorbidity Index | NPR ^a^ |
|  |  |
| ACEi | NPDR: ATC C09AA01, C09AA02, C09AA03, C09AA05, C09BA02, C09BA03, C09BA05 ^c^ |
| ARB | NPDR: ATC C09CA06, C09CA01, C09CA03 ^c^ |
| ARNi | NPDR: ATC C09DX04 ^c^ |
| Beta-blocker | NPDR: ATC C07AB07, C07AG02, C07AB02, C07FB02 ^c^ |
| MRA | NPDR: ATC C03DA01, C03DA04, C03DA05 ^c^ |
| SGLT2i | NPDR: ATC A10BK01, A10BK03 ^c^ |
| Ivabradin | NPDR: ATC C01EB17 ^c^ |
| Loop diuretics | NPDR: ATC C03C ^c^ |
| Digoxin | NPDR: ATC C01AA05 ^c^ |
| Nitrate | NPDR: ATC C01DA ^c^ |
| Number of other CV and Non-CV medications | NPDR: Number of unique ATC codes, excluding GDMT, on 2nd level (therapeutic subgroup) ^c^ |
| Device therapy | SwedeHF |
| Family situation | LISA |
| Marital status | LISA |
| Children | RTP |
| Education | LISA |
| Disposable income | LISA |
| EQ5D-vas | SwedeHF |
| CV mortality | CDR: ICD-10 I, J81, K761, R570, G45 ^e^ |
| HF hospitalization | NPR: ICD-10 I110, I130, I132, I255, I420, I423, I425, I426, I427, I428, I429, I43, I50, J81, K761, R570 ^d^ |

^a^ Derived from diagnoses in all positions, in-patient and out-patient specialty care within 10 years prior to index using ICD-9/10 according to Ludvigsson, 2021.^8^

^b^ Derived from diagnoses/operations in all positions, in-patient and out-patient specialty care within 5 years prior to index (ICD-10)/1987 (ICD-9) unless stated otherwise.

^c^ Medications derived from prescriptions within 4 months prior to index up until 14 days after index unless stated otherwise.

^d^ Hospitalizations are derived from diagnoses in main position, in-patient care.

^e^ Cause of death is derived from the underlying cause of death.

**Abbreviations:** ACEi, angiotensin-converting enzyme inhibitor; ARB, angiotensin receptor blocker; ARNi, angiotensin receptor–neprilysin inhibitor; ATC, Anatomical Therapeutic Chemical; BMI, body mass index; CDR, Cause of Death Register; CKD-EPI, Chronic Kidney Disease Epidemiology Collaboration; COPD, chronic obstructive pulmonary disease; CV, cardiovascular; DR, dialysis-related procedure code; eGFR, estimated glomerular filtration rate; EQ5D-vas, EuroQol 5-Dimensions visual analogue scale; EF, ejection fraction; GDMT, guideline-directed medical therapy; Hb, hemoglobin; HF, heart failure; HFH, heart failure hospitalization; ICD, International Classification of Diseases; ICD-9, International Classification of Diseases, Ninth Revision; ICD-10, International Classification of Diseases, Tenth Revision; ICD (device), implantable cardioverter-defibrillator; LISA, Longitudinal Integration Database for Health Insurance and Labour Market Studies; LVEF, left ventricular ejection fraction; MRA, mineralocorticoid receptor antagonist; NPR, National Patient Register; NPDR, National Prescribed Drug Register; NT-proBNP, N-terminal pro–B-type natriuretic peptide; PIN, personal identification number; RTP, Register of the Total Population; SCB, Statistics Sweden; SGLT2i, sodium–glucose cotransporter 2 inhibitor; SwedeHF, Swedish Heart Failure Registry; TIA, transient ischemic attack

### Table S5. Proportion of patients with good adherence and 1-year persistence according to recent HF hospitalization status.

|  | **Beta-blocker** | **ACEi/ARB/ARNi** | **MRA** | **SGLT2i** |
| --- | --- | --- | --- | --- |
| **Good adherence (PDC ≥80%)** | | | | |
| HF hospitalization in the past 3 months | 94% | 94% | 89% | 94% |
| No HF hospitalization in the past 3 months | 95% | 95% | 90% | 94% |
| **1-year persistence** | | | | |
| HF hospitalization in the past 3 months | 90% | 88% | 75% | 86% |
| No HF hospitalization in the past 3 months | 90% | 90% | 78% | 86% |

**Abbreviations:** ACEi, angiotensin-converting enzyme inhibitor; ARNi, angiotensin receptor–neprilysin inhibitor; ARB, angiotensin receptor blocker; HF, heart failure; MRA, mineralocorticoid receptor antagonist; PDC, proportion of days covered; SGLT2i, sodium–glucose cotransporter 2 inhibitor

### Table S6. Independent predictors of patient adherence.

| **Variable** | **Beta-blocker** | **ACEi/ARB/ARNi** | **MRA** | **SGLT2i** |
| --- | --- | --- | --- | --- |
| Adherence ≥ 80%, n (%) | 13448 (95.0) | 13741 (95.4) | 9497 (91.7) | 8853 (94.0) |
| Index year | 1.0 (0.90-1.09), 0.871 | 1.1 (0.97-1.17), 0.211 | 1.3 (1.20-1.43), <0.001 | 1.3 (1.16-1.47), <0.001 |
| Male vs Female | 0.6 (0.49-0.74), <0.001 | 0.6 (0.50-0.75), <0.001 | 0.9 (0.72-1.02), 0.075 | 1.1 (0.90-1.36), 0.340 |
| Age (years) 70-80 vs <70 | 1.2 (0.98-1.46), 0.073 | 1.5 (1.19-1.81), <0.001 | 1.1 (0.89-1.29), 0.489 | 1.4 (1.13-1.77), 0.002 |
| Age (years) >80 vs <70 | 1.3 (1.02-1.75), 0.034 | 1.3 (1.03-1.75), 0.028 | 1.0 (0.76-1.23), 0.800 | 1.4 (1.01-1.81), 0.043 |
| Duration HF (months) ≥6 vs <6 | 0.7 (0.61-0.87), 0.001 | 0.7 (0.58-0.84), <0.001 | 0.9 (0.72-1.02), 0.079 | 0.7 (0.55-0.82), <0.001 |
| Previous HFH within 3 months | 0.9 (0.78-1.14), 0.546 | 0.9 (0.77-1.12), 0.458 | 0.9 (0.76-1.06), 0.207 | 1.0 (0.78-1.18), 0.700 |
| Follow-up referral HF nurse clinic | 1.1 (0.83-1.56), 0.432 | 1.5 (1.12-1.98), 0.007 | 0.9 (0.65-1.23), 0.476 | 1.0 (0.68-1.44), 0.960 |
| Follow-up hospital vs Primary care/Other | 1.0 (0.77-1.33), 0.916 | 1.1 (0.82-1.40), 0.598 | 1.0 (0.79-1.33), 0.864 | 1.2 (0.86-1.62), 0.300 |
| LVEF (%) <30 vs 30-39 | 1.1 (0.94-1.31), 0.232 | 1.4 (1.16-1.65), <0.001 | 1.1 (0.98-1.34), 0.080 | 1.2 (0.97-1.41), 0.101 |
| NYHA class III-IV vs I-II | 0.8 (0.70-1.03), 0.096 | 0.8 (0.64-0.92), 0.006 | 0.9 (0.72-1.02), 0.081 | 0.9 (0.69-1.09), 0.231 |
| BMI (kg/m²) ≥30 vs <30 | 1.1 (0.86-1.31), 0.574 | 1.2 (0.97-1.53), 0.093 | 1.1 (0.90-1.36), 0.328 | 1.2 (0.92-1.47), 0.213 |
| Systolic bloodpressure (mmHg) <100 vs 100-139 | 1.0 (0.74-1.26), 0.800 | 0.8 (0.60-0.99), 0.039 | 0.9 (0.70-1.10), 0.256 | 1.1 (0.81-1.42), 0.641 |
| Systolic bloodpressure (mmHg) ≥140 vs 100-139 | 0.8 (0.68-1.01), 0.061 | 0.9 (0.76-1.18), 0.623 | 0.8 (0.70-1.02), 0.076 | 0.8 (0.67-1.08), 0.174 |
| Heart rate (beats/min) <50 vs 50-70 | 0.3 (0.21-0.43), <0.001 | 1.3 (0.64-2.54), 0.494 | 1.1 (0.65-1.75), 0.809 | 1.3 (0.62-2.70), 0.489 |
| Heart rate (beats/min) >70 vs 50-70 | 0.8 (0.70-0.98), 0.026 | 0.7 (0.60-0.85), <0.001 | 0.8 (0.71-0.96), 0.014 | 0.6 (0.53-0.77), <0.001 |
| eGFR (mL/min/1.73 m²) 30-59 vs ≥60 | 0.9 (0.76-1.11), 0.383 | 0.8 (0.63-0.94), 0.009 | 0.6 (0.47-0.65), <0.001 | 1.0 (0.84-1.28), 0.744 |
| eGFR (mL/min/1.73 m²) <30 vs ≥60 | 1.5 (0.90-2.63), 0.112 | 0.5 (0.33-0.69), <0.001 | 0.3 (0.19-0.40), <0.001 | 0.5 (0.33-0.83), 0.006 |
| Hypokalemia vs Normakalemia | 1.0 (0.59-1.84), 0.879 | 0.8 (0.47-1.21), 0.242 | 1.4 (0.81-2.42), 0.222 | 1.5 (0.78-2.85), 0.232 |
| Hyperkalemia vs Normakalemia | 1.1 (0.70-1.64), 0.750 | 1.2 (0.77-1.83), 0.449 | 0.6 (0.42-0.75), <0.001 | 2.0 (1.08-3.56), 0.027 |
| NT-proBNP (pg/ml) 2nd tertile vs 1st tertile | 1.1 (0.86-1.29), 0.621 | 0.9 (0.74-1.17), 0.517 | 1.1 (0.91-1.39), 0.295 | 0.9 (0.71-1.13), 0.351 |
| NT-proBNP (pg/ml) 3rd tertile vs 1st tertile | 1.1 (0.89-1.45), 0.306 | 0.8 (0.60-0.97), 0.028 | 1.0 (0.75-1.21), 0.693 | 0.8 (0.60-1.04), 0.093 |
| Smoking | 0.8 (0.58-0.99), 0.048 | 0.9 (0.66-1.11), 0.243 | 0.7 (0.57-0.94), 0.016 | 0.9 (0.66-1.21), 0.466 |
| Diabetes | 1.1 (0.93-1.40), 0.196 | 1.1 (0.89-1.34), 0.422 | 0.9 (0.77-1.11), 0.396 | 1.0 (0.85-1.30), 0.659 |
| Hypertension | 1.0 (0.86-1.23), 0.773 | 1.0 (0.87-1.26), 0.643 | 1.0 (0.84-1.19), 0.985 | 1.1 (0.89-1.35), 0.376 |
| Ischemic heart disease | 0.8 (0.68-0.96), 0.017 | 1.0 (0.85-1.21), 0.877 | 1.0 (0.85-1.17), 0.994 | 0.8 (0.67-0.98), 0.034 |
| Peripheral artery disease | 1.0 (0.77-1.33), 0.929 | 0.9 (0.65-1.11), 0.235 | 1.2 (0.94-1.60), 0.132 | 0.8 (0.60-1.05), 0.104 |
| Atrial fibrillation/flutter | 1.1 (0.97-1.35), 0.122 | 1.3 (1.09-1.55), 0.003 | 1.1 (0.92-1.26), 0.350 | 1.3 (1.06-1.55), 0.011 |
| Stroke | 0.9 (0.74-1.19), 0.599 | 1.0 (0.77-1.25), 0.854 | 1.2 (0.93-1.46), 0.177 | 1.1 (0.82-1.40), 0.633 |
| Anemia | 0.8 (0.67-0.99), 0.037 | 0.9 (0.71-1.06), 0.168 | 0.8 (0.63-0.90), 0.002 | 0.8 (0.67-1.05), 0.123 |
| Valvular disease | 0.8 (0.62-0.92), 0.005 | 0.9 (0.74-1.12), 0.398 | 1.0 (0.86-1.26), 0.699 | 0.9 (0.70-1.09), 0.238 |
| COPD | 0.9 (0.73-1.22), 0.648 | 0.8 (0.65-1.06), 0.140 | 0.8 (0.67-1.07), 0.158 | 0.8 (0.62-1.06), 0.122 |
| Malignant cancer within 3 years | 0.9 (0.70-1.12), 0.322 | 0.9 (0.70-1.13), 0.336 | 0.9 (0.70-1.08), 0.213 | 0.8 (0.61-1.01), 0.058 |
| Muscoloskeletal/connective tissue diseases within 3 years | 0.9 (0.77-1.11), 0.391 | 0.9 (0.78-1.13), 0.487 | 0.8 (0.72-0.99), 0.040 | 1.0 (0.81-1.21), 0.936 |
| Alcohol abuse | 0.6 (0.47-0.86), 0.004 | 0.6 (0.47-0.87), 0.004 | 0.6 (0.48-0.87), 0.004 | 0.9 (0.58-1.29), 0.471 |
| 2-4 vs 0-1 other medications | 1.2 (0.89-1.64), 0.229 | 1.4 (1.03-1.90), 0.034 | 0.8 (0.59-1.21), 0.353 | 0.9 (0.56-1.32), 0.491 |
| 5-6 vs 0-1 other medications | 1.5 (1.04-2.06), 0.028 | 1.7 (1.23-2.44), 0.002 | 1.0 (0.65-1.40), 0.815 | 0.9 (0.60-1.47), 0.780 |
| 7-8 vs 0-1 other medications | 1.7 (1.18-2.50), 0.005 | 1.9 (1.28-2.69), 0.001 | 1.0 (0.64-1.42), 0.815 | 0.8 (0.52-1.34), 0.456 |
| ≥9 vs 0-1 other medications | 2.0 (1.33-2.97), 0.001 | 2.1 (1.40-3.08), <0.001 | 1.2 (0.77-1.80), 0.440 | 1.1 (0.69-1.88), 0.613 |
| CRT/ICD | 1.2 (0.93-1.46), 0.189 | 1.0 (0.81-1.28), 0.906 | 0.9 (0.73-1.08), 0.230 | 1.1 (0.84-1.38), 0.544 |
| Living alone vs Cohabitating | 0.8 (0.72-1.00), 0.049 | 0.6 (0.52-0.73), <0.001 | 0.9 (0.75-1.02), 0.095 | 1.0 (0.85-1.22), 0.846 |
| Children | 1.0 (0.78-1.18), 0.673 | 1.1 (0.92-1.38), 0.232 | 1.1 (0.95-1.39), 0.160 | 1.1 (0.83-1.33), 0.676 |
| Secondary vs Compulsory school | 0.9 (0.77-1.11), 0.409 | 1.1 (0.92-1.34), 0.264 | 0.9 (0.78-1.10), 0.357 | 1.1 (0.90-1.37), 0.332 |
| University vs Compulsory school | 0.9 (0.72-1.13), 0.375 | 1.1 (0.84-1.33), 0.620 | 0.8 (0.66-1.00), 0.053 | 0.8 (0.65-1.05), 0.117 |
| Income 2nd tertile vs 1st tertile | 1.3 (1.04-1.53), 0.016 | 1.4 (1.15-1.68), 0.001 | 1.3 (1.08-1.53), 0.004 | 1.1 (0.88-1.32), 0.480 |
| Income 3rd tertile vs 1st tertile | 1.4 (1.14-1.72), 0.001 | 1.7 (1.35-2.08), <0.001 | 1.6 (1.31-1.93), <0.001 | 1.7 (1.33-2.14), <0.001 |

**Abbreviations:** ACEi, angiotensin-converting enzyme inhibitor; ARNi, angiotensin receptor–neprilysin inhibitor; ARB, angiotensin receptor blocker; BMI, body mass index; COPD, chronic obstructive pulmonary disease; CRT, cardiac resynchronization therapy; eGFR, estimated glomerular filtration rate; HF, heart failure; HFH, heart failure hospitalization; HR, hazard ratio; ICD, implantable cardioverter-defibrillator; LVEF, left ventricular ejection fraction; MRA, mineralocorticoid receptor antagonist; NT-proBNP, N-terminal pro–B-type natriuretic peptide; NYHA, New York Heart Association; SGLT2i, sodium–glucose cotransporter 2 inhibitor

### Table S7. Independent predictors of 1-year patient persistence.

| **Variable** | **Beta-blocker** | **ACEi/ARB/ARNi** | **MRA** | **SGLT2i** |
| --- | --- | --- | --- | --- |
| Persistance, n (%) | 6333 (89.9) | 6496 (90.1) | 3952 (79.2) | 3049 (85.9) |
| Index year | 0.9 (0.78-1.07), 0.278 | 1.0 (0.83-1.14), 0.768 | 1.1 (0.96-1.28), 0.145 | 1.1 (0.89-1.37), 0.356 |
| Male vs Female | 0.8 (0.63-0.94), 0.009 | 0.7 (0.59-0.88), 0.001 | 0.9 (0.78-1.09), 0.354 | 0.9 (0.73-1.18), 0.535 |
| Age (years) 70-80 vs <70 | 1.0 (0.84-1.26), 0.767 | 1.1 (0.90-1.35), 0.364 | 0.9 (0.76-1.09), 0.296 | 1.2 (0.96-1.57), 0.109 |
| Age (years) >80 vs <70 | 1.0 (0.75-1.29), 0.915 | 1.0 (0.76-1.29), 0.939 | 0.8 (0.64-1.03), 0.088 | 1.1 (0.80-1.55), 0.526 |
| Duration HF (months) ≥6 vs <6 | 1.0 (0.84-1.22), 0.887 | 0.9 (0.79-1.14), 0.558 | 1.0 (0.88-1.22), 0.676 | 1.0 (0.80-1.26), 0.963 |
| Previous HFH within 3 months | 0.9 (0.75-1.08), 0.268 | 0.9 (0.72-1.04), 0.112 | 1.0 (0.81-1.12), 0.569 | 1.0 (0.78-1.23), 0.879 |
| Follow-up referral HF nurse clinic | 1.1 (0.76-1.52), 0.698 | 1.3 (0.98-1.82), 0.063 | 0.9 (0.63-1.18), 0.362 | 1.0 (0.67-1.58), 0.891 |
| Follow-up hospital vs Primary care/Other | 0.7 (0.51-0.96), 0.029 | 1.1 (0.84-1.42), 0.518 | 1.0 (0.77-1.30), 0.972 | 1.1 (0.74-1.60), 0.674 |
| LVEF (%) <30 vs 30-39 | 1.1 (0.92-1.30), 0.326 | 1.3 (1.09-1.56), 0.003 | 1.0 (0.89-1.20), 0.670 | 1.2 (0.95-1.45), 0.143 |
| NYHA class III-IV vs I-II | 0.8 (0.64-0.96), 0.019 | 0.8 (0.69-1.00), 0.052 | 0.9 (0.75-1.04), 0.138 | 1.1 (0.84-1.37), 0.564 |
| BMI (kg/m²) ≥30 vs <30 | 1.0 (0.82-1.27), 0.847 | 1.0 (0.78-1.18), 0.691 | 1.1 (0.90-1.29), 0.404 | 1.0 (0.79-1.30), 0.928 |
| Systolic bloodpressure (mmHg) <100 vs 100-139 | 0.9 (0.67-1.20), 0.471 | 0.8 (0.60-1.04), 0.095 | 0.8 (0.59-0.96), 0.020 | 0.8 (0.62-1.13), 0.245 |
| Systolic bloodpressure (mmHg) ≥140 vs 100-139 | 0.9 (0.75-1.12), 0.383 | 1.0 (0.80-1.21), 0.858 | 1.0 (0.80-1.18), 0.789 | 1.1 (0.84-1.44), 0.502 |
| Heart rate (beats/min) <50 vs 50-70 | 0.3 (0.23-0.48), <0.001 | 0.8 (0.50-1.35), 0.437 | 0.9 (0.56-1.34), 0.516 | 1.2 (0.61-2.26), 0.628 |
| Heart rate (beats/min) >70 vs 50-70 | 1.0 (0.86-1.22), 0.777 | 0.8 (0.71-1.00), 0.050 | 0.9 (0.78-1.05), 0.178 | 0.8 (0.64-0.96), 0.021 |
| eGFR (mL/min/1.73 m²) 30-59 vs ≥60 | 1.1 (0.87-1.30), 0.563 | 0.8 (0.64-0.94), 0.010 | 0.6 (0.52-0.74), <0.001 | 0.9 (0.75-1.21), 0.663 |
| eGFR (mL/min/1.73 m²) <30 vs ≥60 | 1.3 (0.73-2.24), 0.389 | 0.6 (0.39-0.92), 0.021 | 0.3 (0.20-0.54), <0.001 | 0.7 (0.33-1.33), 0.247 |
| Hypokalemia vs Normakalemia | 0.7 (0.39-1.10), 0.107 | 0.7 (0.41-1.11), 0.119 | 0.8 (0.48-1.32), 0.387 | 1.0 (0.49-2.07), 0.996 |
| Hyperkalemia vs Normakalemia | 1.1 (0.73-1.78), 0.570 | 0.9 (0.61-1.33), 0.602 | 0.5 (0.37-0.69), <0.001 | 0.5 (0.35-0.85), 0.007 |
| NT-proBNP (pg/ml) 2nd tertile vs 1st tertile | 1.0 (0.82-1.27), 0.861 | 1.1 (0.89-1.38), 0.376 | 1.1 (0.90-1.31), 0.394 | 1.1 (0.86-1.43), 0.437 |
| NT-proBNP (pg/ml) 3rd tertile vs 1st tertile | 1.2 (0.90-1.48), 0.251 | 1.0 (0.77-1.28), 0.961 | 1.1 (0.86-1.36), 0.528 | 1.0 (0.72-1.33), 0.885 |
| Smoking | 1.1 (0.84-1.54), 0.403 | 0.9 (0.71-1.26), 0.699 | 0.8 (0.59-0.96), 0.021 | 0.9 (0.66-1.30), 0.674 |
| Diabetes | 1.2 (0.95-1.46), 0.128 | 1.2 (0.97-1.47), 0.089 | 1.0 (0.80-1.14), 0.627 | 1.2 (0.96-1.55), 0.112 |
| Hypertension | 1.0 (0.82-1.18), 0.879 | 1.0 (0.79-1.16), 0.655 | 0.9 (0.77-1.08), 0.292 | 1.0 (0.78-1.24), 0.898 |
| Ischemic heart disease | 1.0 (0.86-1.23), 0.747 | 1.1 (0.96-1.37), 0.140 | 1.1 (0.94-1.30), 0.209 | 1.1 (0.85-1.32), 0.613 |
| Peripheral artery disease | 1.1 (0.80-1.51), 0.566 | 0.9 (0.70-1.25), 0.645 | 1.1 (0.85-1.47), 0.418 | 0.7 (0.48-0.93), 0.017 |
| Atrial fibrillation/flutter | 1.0 (0.84-1.18), 0.980 | 1.0 (0.86-1.21), 0.856 | 0.9 (0.77-1.05), 0.165 | 1.2 (0.93-1.42), 0.187 |
| Stroke | 0.9 (0.71-1.17), 0.461 | 1.3 (1.02-1.75), 0.033 | 1.3 (1.02-1.61), 0.036 | 1.3 (0.95-1.80), 0.100 |
| Anemia | 0.9 (0.70-1.08), 0.204 | 0.9 (0.69-1.05), 0.128 | 0.7 (0.61-0.87), <0.001 | 0.8 (0.63-1.06), 0.134 |
| Valvular disease | 1.0 (0.77-1.18), 0.645 | 1.0 (0.84-1.29), 0.690 | 1.0 (0.83-1.21), 0.997 | 0.9 (0.69-1.14), 0.339 |
| COPD | 0.9 (0.66-1.14), 0.302 | 0.9 (0.68-1.14), 0.343 | 0.9 (0.72-1.15), 0.421 | 1.0 (0.72-1.41), 0.976 |
| Malignant cancer within 3 years | 1.1 (0.85-1.46), 0.433 | 0.8 (0.64-1.03), 0.086 | 1.4 (1.07-1.73), 0.012 | 1.0 (0.71-1.34), 0.880 |
| Muscoloskeletal/connective tissue diseases within 3 years | 1.0 (0.82-1.19), 0.863 | 0.9 (0.74-1.06), 0.191 | 0.9 (0.78-1.08), 0.298 | 0.9 (0.69-1.09), 0.214 |
| Alcohol abuse | 0.8 (0.56-1.16), 0.248 | 0.8 (0.54-1.10), 0.149 | 0.8 (0.59-1.14), 0.239 | 1.0 (0.61-1.51), 0.855 |
| 2-4 vs 0-1 other medications | 1.4 (1.03-1.87), 0.032 | 1.2 (0.87-1.68), 0.255 | 0.9 (0.65-1.27), 0.583 | 1.3 (0.88-1.97), 0.180 |
| 5-6 vs 0-1 other medications | 1.5 (1.07-2.07), 0.018 | 1.1 (0.77-1.56), 0.618 | 0.9 (0.63-1.28), 0.547 | 1.3 (0.81-1.93), 0.307 |
| 7-8 vs 0-1 other medications | 1.7 (1.20-2.51), 0.004 | 1.1 (0.78-1.68), 0.488 | 0.8 (0.56-1.18), 0.276 | 1.3 (0.80-2.05), 0.312 |
| ≥9 vs 0-1 other medications | 1.8 (1.18-2.62), 0.005 | 1.0 (0.69-1.54), 0.874 | 0.9 (0.60-1.32), 0.560 | 1.2 (0.70-1.91), 0.562 |
| CRT/ICD | 1.4 (1.10-1.83), 0.007 | 1.3 (1.00-1.63), 0.050 | 1.0 (0.82-1.23), 0.964 | 1.0 (0.74-1.27), 0.814 |
| Living alone vs Cohabitating | 1.0 (0.85-1.20), 0.917 | 0.9 (0.73-1.03), 0.101 | 0.9 (0.82-1.10), 0.501 | 1.0 (0.79-1.19), 0.751 |
| Children | 1.1 (0.85-1.32), 0.597 | 1.0 (0.81-1.25), 0.948 | 1.1 (0.87-1.30), 0.541 | 1.0 (0.74-1.27), 0.829 |
| Secondary vs Compulsory school | 0.9 (0.76-1.11), 0.380 | 1.1 (0.92-1.33), 0.289 | 1.0 (0.86-1.21), 0.811 | 0.9 (0.71-1.13), 0.355 |
| University vs Compulsory school | 0.9 (0.68-1.08), 0.184 | 1.0 (0.81-1.27), 0.914 | 1.0 (0.81-1.22), 0.946 | 0.8 (0.63-1.12), 0.234 |
| Income 2nd tertile vs 1st tertile | 1.2 (0.96-1.43), 0.127 | 1.5 (1.23-1.83), <0.001 | 0.9 (0.79-1.12), 0.487 | 1.0 (0.80-1.29), 0.928 |
| Income 3rd tertile vs 1st tertile | 1.2 (0.97-1.49), 0.085 | 1.4 (1.15-1.75), 0.001 | 1.2 (1.02-1.49), 0.033 | 1.4 (1.11-1.87), 0.006 |

**Abbreviations:** ACEi, angiotensin-converting enzyme inhibitor; ARNi, angiotensin receptor–neprilysin inhibitor; ARB, angiotensin receptor blocker; BMI, body mass index; COPD, chronic obstructive pulmonary disease; CRT, cardiac resynchronization therapy; eGFR, estimated glomerular filtration rate; HF, heart failure; HFH, heart failure hospitalization; HR, hazard ratio; ICD, implantable cardioverter-defibrillator; LVEF, left ventricular ejection fraction; MRA, mineralocorticoid receptor antagonist; NT-proBNP, N-terminal pro–B-type natriuretic peptide; NYHA, New York Heart Association; SGLT2i, sodium–glucose cotransporter 2 inhibitor

### Table S8. Crude and adjusted associations between number of GDMT components and time to the composite of first HF hospitalization and cardiovascular death.

|  | **Crude HR (95% CI), p** | **Adjusted HR (95% CI), p** |
| --- | --- | --- |
| **2016-2023 (main analysis)** | | |
| No therapy | Reference | Reference |
| Monotherapy | 0.81 (0.70-0.95), 0.008 | 0.74 (0.63-0.87), <0.001 |
| Dual therapy | 0.57 (0.50-0.66), <0.001 | 0.64 (0.55-0.74), <0.001 |
| Triple therapy | 0.54 (0.47-0.62), <0.001 | 0.63 (0.54-0.73), <0.001 |
| Quadruple therapy | 0.36 (0.30-0.41), <0.001 | 0.57 (0.48-0.67), <0.001 |
| **2021-2023 (sensitivity analysis)** | | |
| No therapy | Reference | Reference |
| Monotherapy | 0.81 (0.55-1.19), 0.273 | 0.64 (0.42-0.95), 0.028 |
| Dual therapy | 0.64 (0.45-0.91), 0.012 | 0.61 (0.42-0.89), 0.010 |
| Triple therapy | 0.56 (0.40-0.79), 0.001 | 0.58 (0.40-0.83), 0.003 |
| Quadruple therapy | 0.48 (0.34-0.68), <0.001 | 0.54 (0.37-0.78), 0.001 |

**Abbreviations:** CI, confidence interval; GDMT, guideline-directed medical therapy; HF, heart failure; HR, hazard ratio

### References

1.	Ludvigsson JF, Almqvist C, Bonamy AK, Ljung R, Michaelsson K, Neovius M, et al. Registers of the Swedish total population and their use in medical research. *Eur J Epidemiol* 2016;**31**:125-136. doi: <https://doi.org/10.1007/s10654-016-0117-y>

2.	Ludvigsson JF, Svedberg P, Olén O, Bruze G, Neovius M. The longitudinal integrated database for health insurance and labour market studies (LISA) and its use in medical research. *Eur J Epidemiol* 2019;**34**:423-437. doi: <https://doi.org/10.1007/s10654-019-00511-8>

3.	Ludvigsson JF, Andersson E, Ekbom A, Feychting M, Kim JL, Reuterwall C, et al. External review and validation of the Swedish national inpatient register. *BMC Public Health* 2011;**11**:450. doi: <https://doi.org/10.1186/1471-2458-11-450>

4.	Brooke HL, Talbäck M, Hörnblad J, Johansson LA, Ludvigsson JF, Druid H, et al. The Swedish cause of death register. *Eur J Epidemiol* 2017;**32**:765-773. doi: <https://doi.org/10.1007/s10654-017-0316-1>

5.	Wettermark B, Hammar N, Fored CM, Leimanis A, Otterblad Olausson P, Bergman U, et al. The new Swedish Prescribed Drug Register--opportunities for pharmacoepidemiological research and experience from the first six months. *Pharmacoepidemiol Drug Saf* 2007;**16**:726-735. doi: <https://doi.org/10.1002/pds.1294>

6.	Savarese G, Vasko P, Jonsson A, Edner M, Dahlstrom U, Lund LH. The Swedish Heart Failure Registry: a living, ongoing quality assurance and research in heart failure. *Ups J Med Sci* 2019;**124**:65-69. doi: <https://doi.org/10.1080/03009734.2018.1490831>

7.	Inker LA, Eneanya ND, Coresh J, Tighiouart H, Wang D, Sang Y, et al. New Creatinine- and Cystatin C-Based Equations to Estimate GFR without Race. *N Engl J Med* 2021;**385**:1737-1749. doi: <https://doi.org/10.1056/NEJMoa2102953>

8.	Ludvigsson JF, Appelros P, Askling J, Byberg L, Carrero JJ, Ekström AM, et al. Adaptation of the Charlson Comorbidity Index for Register-Based Research in Sweden. *Clin Epidemiol* 2021;**13**:21-41. doi: <https://doi.org/10.2147/clep.s282475>
